# Synopsis of Schizopteridae (Hemiptera, Heteroptera, Dipsocoromorpha) from the United States, with description of seven new species from the US and Mexico

**DOI:** 10.3897/zookeys.796.24176

**Published:** 2018-11-15

**Authors:** Christiane Weirauch, Rochelle Hoey-Chamberlain, Alexander Knyshov

**Affiliations:** 1 Department of Entomology, University of California, Riverside, Riverside, CA 92521, USA University of California Riverside United States of America

**Keywords:** biodiversity, minute litter bug, Nearctic region, systematics, true bug, taxonomy

## Abstract

Because species diversity of the small true bug family Schizopteridae is greatest in tropical and subtropical areas, it is not surprising that only four species have been described from the United States. As part of a larger project on the taxonomy and phylogenetics of Schizopteridae, 178 specimens from the United States were examined. This material contained representatives of the previously described species *Glyptocombussaltator* Heidemann, 1906, *Corixideamajor* McAtee & Malloch, 1925, *Nannocorisarenarius* Blatchley, 1926, and *Schizopterabispina* McAtee & Malloch, 1925, but also six undescribed species. These new taxa are described as *Glyptocombushalbertae***sp. n.**, *Glyptocombussuteri***sp. n.**, *Nannocorisanophorus***sp. n.**, *Nannocorisbrevipilus***sp. n.**, Schizoptera (Cantharocoris) rileyi**sp. n.**, and Schizoptera (Schizoptera) henryi**sp. n.** Habitus images and genitalic illustrations of the previously described and the new species are provided as well as a map showing distribution ranges of these species in the United States and Mexico. To provide a comprehensive treatment of the small genus *Glyptocombus* Heidemann, 1906, *Glyptocombusmexicanus***sp. n**. is also described that, to our knowledge, occurs only in Mexico, and the female of one additional undescribed *Glyptocombus* species is documented from Mexico.

## Introduction

The small true bug family Schizopteridae (Hemiptera: Heteroptera) in the infraorder Dipsocoromorpha contains approximately 355 described species (Emsley 1969, Hill 1984, McAtee and Malloch 1925, Weirauch et al. 2018, Wygodzinsky 1955). Although several species occur in temperate regions in Japan, Tasmania, and New Zealand, the great majority of species have been described from wet tropical and subtropical areas around the globe (Emsley 1969, Knyshov et al. 2016, Wygodzinsky 1951). The fauna of Schizopteridae in the United States could therefore be expected to be relatively small. Consistent with this prediction, only four species representing four genera were described during the first quarter of the 20^th^ century and these are currently the only species known to occur in the United States (Henry 1988, 2010). The four species appear to have relatively large distribution ranges in the eastern and southeastern United States including one that was originally described from Guatemala (Henry et al. 2010, Allen and Carlton 1989, Heidemann 1906, Hoffman et al. 2005, Roble and Hoffman 2000, Hoffman et al. 2007, McAtee and Malloch 1925).

Heidemann (1906) described the monotypic genus *Glyptocombus* Heidemann, 1906 to accommodate *Glyptocombussaltator* Heidemann, 1906 described from specimens collected on Plummers Island in Maryland. This species was subsequently recorded from Arkansas, the District of Columbia, Michigan, Tennessee, Virginia and Washington DC (Henry 1988, Allen and Carlton 1989, Roble and Hoffman 2000). Heidemann (1906) noted the resemblance of this species to *Hypselosoma* Reuter and it is classified in the Hypselosomatinae (Emsley 1969). It has remained one of only a handful of New World genera in this subfamily (Uhler 1894, Emsley 1969, Carpintero and Dellapé 2006).

The three remaining species of Schizopteridae recorded from the US belong to the Schizopterinae and are classified in the speciose genera *Corixidea* Reuter, 1891, *Nannocoris* Reuter, 1891, and *Schizoptera* Fieber, 1860. Distributions of species in the three genera range from the southern parts of South America to the US. In addition to the currently described species (9 in *Corixidea*, 12 in *Nannocoris*, and ~80 in *Schizoptera*: Emsley 1969, Leon and Weirauch 2016a,b), we examined specimens representing a large number of undescribed species from across the New World during taxonomic revisions that are ongoing (Weirauch Lab, unpublished data). *Corixideamajor* McAtee & Malloch, 1925 was described from Clarksville in Tennessee and has been recorded from Florida, Virginia, Arkansas and Oklahoma (Hoffman et al. 2005, Henry et al. 2010). *Nannocorisarenarius* Blatchley, 1926 is known from Georgia, North Carolina, and Virginia (Hoffman et al. 2007), but was originally described from Florida (Blatchley 1926). Neither species is known to occur outside the US and both are clearly differentiated from congeneric species in Central America by head shape, wing type and venation (*Nannocoris*) and size, coloration and male genitalic structures (*Corixidea*). The situation is less clear for *Schizopterabispina* McAtee & Malloch, 1925 that was originally described from Guatemala and recorded from Mexico (McAtee and Malloch 1925) and subsequently reported from Florida (Blatchley 1926). McAtee and Malloch (1925) indicated that the lateral spines on the male subgenital plate were shorter in the Mexican specimen compared to the holotype, but nevertheless treated them as conspecific. The species was originally classified in the subgenus Schizoptera (Lophopleurum) that contained six additional species from Central America and Trinidad (McAtee and Malloch 1925, Emsley 1969, Costas et al. 2015). Based on the lack of reciprocal monophyly and diagnostic features, Schizoptera (Lophopleurum) was recently synonymized with Schizoptera (Cantharocoris) (Leon and Weirauch 2017) that contains an additional six species from the Caribbean, Central and Northern South America including Trinidad (McAtee and Malloch 1925, Emsley 1969). Species in the subgenus Schizoptera (Cantharocoris) are recognized by the glabrous area associated with the scent gland groove extending to or beyond the midline of the metapleuron; in species of Schizoptera (Schizoptera) the glabrous area is restricted to the ventral margin of the scent gland groove.

We here provide an updated synopsis of the Schizopteridae occurring in the United States. We have examined >8,000 specimens of New World Schizopteridae as part of a project on the biodiversity and systematics of this group. The majority of specimens were curated from ethanol-preserved residues of passive trap samples, but we also borrowed point and card-mounted specimens from various collections. The 178 U.S. specimens of Schizopteridae that we have located and examined are mostly point-mounted and were borrowed from seven collections. Texas A&M and the Florida State Collection of Arthropods provided the bulk of the material, with 89 and 53 specimens, respectively. We also examined specimens of *Corixidea*, *Nannocoris*, and Schizoptera (Cantharocoris) from Mexico and other countries in Central America to assure that our synopsis of previously described and new taxa treated in this paper is as thorough as possible with respect to distribution ranges that extend beyond the administrative borders of the U.S. Additional undescribed species of the three schizopterine genera from Mexico and Central America will be treated as part of separate, genus-focused publications. In contrast, the revision of *Glyptocombus* presented as part of this study is comprehensive based on the available material and includes a new species known only from Mexico. We here provide a synopsis of the ten species of Schizopteridae that occur in the US, describe six of them as new, provide habitus images and genitalic illustrations of previously described and new species, and document their distribution ranges.

## Materials and method

We have examined approximately 8,000 specimens of Schizopteridae from the Nearctic and Neotropical regions as part of a US National Science Foundation project on the biodiversity and systematics of the true bug infraorder Dipsocoromorpha. Among these were 178 specimens from the United States, representing the four previously described species and several undescribed species in the genera *Glyptocombus*, *Corixidea*, *Nannocoris*, and *Schizoptera*. We surveyed point-mounted specimens belonging to these four genera from Mexico and other Central American countries as well as the Caribbean to ensure that this synopsis includes relevant material from outside the boundaries of the United States. Natural history collection acronyms are as follows:

**ABS**Archbold Biological Station, Lake Placid, Florida, USA;

**AMNH**American Museum of Natural History, New York, USA;

**NHMUK**Natural History Museum, London, UK;

**FMNH**The Field Museum of Natural History, Chicago, USA;

**FSCA**Florida State Collection of Arthropods, Gainesville, USA;

**NCSU**North Carolina State University Insect Collection, Raleigh, USA;

**PURC**Purdue Entomological Research Collection, West Lafayette, USA;

**TAMU**Texas A&M University, College Station, USA;

**UCR**Entomology Research Museum, University of California, Riverside, USA;

**USNM**National Museum of Natural History, Washington, D.C., USA;

**VMNH**Virginia Museum of Natural History, Martinsville, USA.

### Imaging, dissections, measurements, databasing, and distribution maps

Habitus images were taken using a Leica DFC 450 C Microsystems system (Leica, Wetzlar, Germany) with a Planapo 1.0× objective. Images of selected morphological characters were produced on the same system with a Planapo 2.0× objective. Individual images were combined using the Leica Application Suite V4.3 software or Zerene stacker V1.02 (Zerene Systems). Images were edited and assembled into image plates in Adobe Photoshop CS4 or CC2018.

To document male and female genitalia, the abdomen was separated from the body and cleared in hot 10% KOH. The male abdomen was temporarily mounted in glycerin on microscope slides, while the female abdomen was stained using Chlorazol Black E for 3 intervals of 30 seconds before slide mounting. Line drawings were prepared using a Nikon Eclipse 80i compound microscope (Nikon, Tokyo, Japan) with camera lucida.

Measurements are in mm (see Table 1; selected measurements and ratios are in text) were taken from the habitus images. Total length was measured from the anterior tip of the head to the tip of the apex of forewing, body length was measured from the anterior tip of the head to the apex of the abdomen, and width between eyes was measured in frontal perspective.

Unique specimen identifier (USI) matrix code labels with prefix and eight-digit number were associated with each specimen. Specimen information was databased using the American Museum of Natural History’s Arthropod Easy Capture (AEC) database (http://www.research.amnh.org/pbi/locality/index.php). Specimen information is also available through the Heteroptera Species Pages (http://research.amnh.org/pbi/heteropteraspeciespage/) and the http://www.discoverlife.org/ website. Maps were prepared using SimpleMappr (http://www.simplemappr.net/) from coordinates exported from the AEC database and edited using Photoshop CS4.

**Table 1.** Measurements of species of *Glyptocombus*, *Corixidea*, *Nannocoris*, and *Schizoptera* in mm.

Taxon		USI	Total length	Body length (ventral)	Head width across eyes	Synthlipsis	Pronotal width	Width forewings	Pronotum length	Pronotal collar length	LC:LP	Width between eyes	Fore femora depth/ height	Fore femora length	DFF:LFF	Hind tibia length	LHT:WP	a3 length	a4 length	a3:a4
* G. halberti *	♂	UCR_ENT 00012022	1.23	0.74	0.67	0.34	0.59	na	0.2	0.05	0.23	0.26	0.16	0.04	0.24	0.52	0.88	0.16	0.1	1.66
* G. mexicanus *	♂♂	UCR_ENT 00094271	1.74	1.74	0.72	0.38	0.58	na	0.24	0.08	0.32	0.31	0.09	0.37	0.23	0.63	1.09	0.31	0.34	0.9
		UCR_ENT 00094275	1.18	1.18	0.75	0.41	0.62	na	0.21	0.07	0.3	0.32	0.09	0.44	0.2	0.69	1.12	0.34	0.35	0.96
	♀	UCR_ENT 00094272	1.51	1.51	0.72	0.38	0.64	na	0.17	0.05	0.28	0.3	0.09	0.36	0.26	0.64	1	0.34	0.33	1.03
* G. saltator *	♂♂	UCR_ENT 00090440_ED5195	1.52	1.52	0.72	0.38	0.57	na	0.18	0.06	0.33	0.27	0.09	0.4	0.23	0.66	1.16			
		UCR_ENT 00094270	1.14	1.04	0.74	0.39	0.6	na	0.23	0.06	0.25	0.3	0.08	0.38	0.21	0.56	0.94			
		UCR_ENT 00094273	1.2	0.93	0.74	0.39	0.61	na	0.21	0.05	0.25	0.31	0.09	0.47	0.2	0.64	1.04			
		UCR_ENT 00011915			0.74	0.4	0.61	na	0.15	0.05	0.35	0.29	0.1	0.44	0.22	0.68				
	♀♀	UCR_ENT 00090439_ED5194	1.49	1.49	0.79	0.41	0.64	na	0.17	0.04	0.26	0.31	0.06	0.41	0.14	0.56	0.88			
		UCR_ENT 00090441_ED5196	1.46	1.46	0.75	0.38	0.62	na	0.2	0.06	0.29	0.28	0.08	0.35	0.24	0.67	1.09			
* G. suteri *	♂	UCR_ENT 00090443_ED5198	1.47	1.47	0.7	0.36	0.57	na	0.25	0.07	0.26	0.27	0.09	0.34	0.26	0.53	0.94	0.33	0.31	1.08
* Corixidea major *	♂♂	UCR_ENT 00093506	1.58	1.12	0.56	0.33	0.66	na												
		UCR_ENT 00012039	1.36	na	0.51	0.3	0.58	na												
	♀	UCR_ENT 00012040	1.42	na	0.53	0.32	0.61	na												
* N. anophorus *	♂	UCR_ENT00094264	1.16	na	0.32	0.22	0.52	na												
* N. arenarius *	♂♂	UCR_ENT 00124097	1.04	na	0.28	0.19	0.42	na												
		UCR_ENT 00120010	1.05	na	0.31	0.2	0.45	0.56												
		UCR_ENT 00124093	1.09	na	0.29	0.18	0.41	0.54												
	♀♀	UCR_ENT 00124094	1.01	na	0.28	0.18	0.42	0.55												
		UCR_ENT 00124095	1.02	na	0.29	0.18	0.41	0.52												
		UCR_ENT 00124096	1	na	0.28	0.19	0.41	0.54												
* N. brevipilus *	♂	UCR_ENT00094257	1.23	na	0.32	0.21	0.52	0.7												
	♀♀	UCR_ENT00094254	1.11	na	0.32	0.2	0.49	0.66												
		UCR_ENT00094250	1.23	na	0.32	0.2	0.48	0.67												
		UCR_ENT00094255	1.14	na	0.32	0.21	0.49	0.63												
S. (C.) bispina	♂	UCR_ENT 000934303	1.28	1.13	0.42	0.25	0.62	na												
S. (C.) rileyi	♂♂	UCR_ENT 00094298	1.42	1.2	0.53	0.31	0.73	0.82												
		UCR_ENT 00093720	1.48	1.13	0.52	0.32	0.71	0.77												
		UCR_ENT 00093725	1.44	1.1	0.51	0.3	0.75	0.82												
		UCR_ENT 00094300	1.44	na	0.49	0.28	0.67	0.76												
	♀♀	UCR_ENT 00093549	1.22	na	0.46	0.3	0.63	0.78												
		UCR_ENT 00093550	1.25	na	0.49	0.32	0.67	0.76												
		UCR_ENT 00094316	1.29	na	0.51	0.32	0.71	0.86												
		UCR_ENT 00094320	1.24	na	0.5	0.33	0.68	0.81												
		UCR_ENT 00093551	1.25	na	0.49	0.31	0.64	0.78												
S. (S.) henryi	♂♂	UCR_ENT 00093649	1.51	1.16	0.5	0.28	0.74	0.87												
		UCR_ENT 00093654	1.67	1.24	0.52	0.26	0.77	0.91												
		UCR_ENT 00093555	1.58	na	0.5	0.26	0.73	0.94												
		UCR_ENT 00093704	1.63	1.31	0.54	0.26	0.79	0.91												

### Nomenclatural acts

This publication and the nomenclatural acts it contains have been registered in ZooBank, the online registration system for the ICZN. The ZooBank LSIDs can be resolved by appending them to the Web address http://zoobank.org/. The LSIDs for nomenclatural acts can be found in corresponding sections of this article.

### Abbreviations and terminology

**ag** anterior gonapophysis;

**An1, An2** first and second anal veins of forewing;

**ano** anophore;

**anop** anophoric process;

**at** anal tube;

**bc** basal cell (cell posterior to R+M);

**bcx** bursa copulatrix;

**ca** conjunctival appendage;

**Cu** cubitus;

**cub** cubital cell (defined as cell bordered anteriorly by distal part of Cu);

**dag** dorsal abdominal gland

**dc** discal cell (defined as cell posterior to M);

**dc1** discal cell 1 (defined as cell posterior to distal part of M);

**g** gonoplac;

**lca** left conjunctival appendage;

**llt9** left laterotergite 9;

**lp** left paramere;

**ovg** opening of vertex gland;

**pc** pronotal collar;

**pg** posterior gonapophysis;

**py** pygophore;

**rc** radial cell removed from wing margin (defined as cell posterior to R or R2+3);

**rc1-3** radial cells along costal margin (defined by cells posterior or distal to R1-R3);

**rca** right conjunctival appendage;

**rlt9** right laterotergite 9;

**rp** right paramere;

**Sc** subcostal vein;

**scc** subcostal cell;

**sp** spiracle;

**spd** spermathecal duct;

**spgl** spermathecal gland;

**spgld** spermathecal gland duct;

**spr** spermathecal reservoir;

**st2-6** sternum 2–6;

**st7** sternum 7 (=subgenital plate in male); sty median styloid;

**t1-9** tergum 1–9;

**t8p** tergum 8 process;

**tc** trapezoidal cell (defined as cell bordered anteriorly by Cu+M);

**v** vesica;

**vp** vesical process.

A larger comparative survey of wing venation across Schizopteridae (Weirauch lab, unpublished data) has indicated that the terminology used in Hypselosomatinae and Schizopterinae is inconsistent. We here introduce a slightly modified terminology for wing veins in Hypselosomatinae from those in Hill (2013) and Hoey-Chamberlain and Weirauch (2016) that also differs from the terminology used for Schizopterinae in Weirauch and Frankenberg (2015) and Leon and Weirauch (2016a,b), but is largely consistent with the terminology for Ogeriinae used by Knyshov et al. (2016). The wing vein terminology is illustrated in Figure 2 for Hypselosomatinae and in Figs 6, 8 for Schizopterinae. More extensive documentation of schizopterid forewing veins is forthcoming as part of a combined morphological and molecular phylogenetic analysis (Knyshov et al., in prep.).

In several recent publications, we have referred to the ventral sclerite of the pregenital abdomen as “sternite,” following, e.g., the terminology used by Emsley (1969). Here we use the terms “tergum” and “sternum” for all dorsal and ventral abdominal sclerites and “laterotergite” for a lateral subdivision of the tergum following recent papers on the morphology of the pregenital abdomen in Dipsocoromorpha (Knyshov et al. 2018) and a putatively closely related group of Heteroptera (Davranoglou et al. 2017).

We follow the genitalic terminology used in recent publications (e.g., Hoey-Chamberlain and Weirauch 2016; Knyshov et al. 2016, 2018; Leon and Weirauch 2016a, b).

## Taxonomy

### Key to Schizopteridae in the United States

**Table:** 

1	Forewing with 4 closed submarginal cells (rc1, rc2, 3, and dc1; Fig. 2A), eyes large, head wider than prothorax, labium 4-segmented (Fig. 1), male sternum 8 well developed (Fig. 3B), ovipositor well developed (Fig. 3C, D)	**2**
–	Forewing with fewer than 4 closed submarginal cells (i.e., rc1 and rc2 fused to rc1-rc2), eyes smaller, head not noticeably wider than prothorax, labium 3-segmented (Figs 4, 6, 8), male sternum 8 reduced (Fig. 9); ovipositor obsolete (Figs 5B, 7G)	**5**
2	Elytriform forewings (Figs 1 [*G.saltator*, *G.mexicanus*], 2B, C)	**3**
–	Macropterous forewings (Figs 1 [*G.halbertae*, *G.suteri*], 2A, D)	**4**
3	Wing veins wider than cells (Fig. 2B), general coloration dark brown to black	***Glyptocombusmexicanus* sp. n.**
–	Veins narrower than cells (Fig. 2C), general coloration reddish brown	***Glyptocombussaltator* Heidemann**
4	General dark coloration (Fig. 1, *G.halbertae*), desclerotized portion of C+Sc vein basal to junction with R1, Cu touching M at proximal corner of dc1, part of Cu distal to tc s-shaped, R2 slightly sigmoid, rc and dcca. as long as bc (Fig. 2A)	***Glyptocombushalbertae* sp. n.**
–	General coloration light brown (Fig. 1, *G.suteri*), C+Sc not desclerotized basal to junction with R1, Cu separated from M by m-cu cross vein, Cu distal to tc straight, R2 straight, rc and dc longer than bc (Fig. 2D)	***Glyptocombussuteri* sp. n.**
5	Labium truncated, pronotal collar absent (Fig. 4)	***Corixideamajor* McAtee and Malloch**
–	Labium tapering, pronotal collar usually present (Figs 6, 8)	**6**
6	Head elongated, labium very slender, posterolateral spine on metepisternum absent, R1 obliquely approaching costal forewing margin (Fig. 6)	**7 (genus *Nannocoris* Reuter)**
–	Head short, labium stout, posterolateral spine on metepisternum well developed, R1 approaching costal forewing margin at right angle (Fig. 8)	**9 (genus *Schizoptera* Fieber)**
7	Forewing veins with long setae (Fig. 6, *N.anophorus*), anophoric process long, reaching anteriorly to terga 5 or 6 (Fig. 7A, D)	***Nannocorisanophorus* sp. n.**
–	Forewing veins with short setae (Fig. 6, *N.arenarius*, *N.brevipilus*), anophoric process short, reaching anteriorly only to tergum 7 (Fig. 7B, C, E, F)	**7**
8	Scutellum with lateral margins of elevated area gently convex (Fig. 6, *N.arenarius*), base of anophoric process slender (Fig. 7B, E)	***Nannocorisarenarius* Blatchley**
–	Scutellum with lateral margins of elevated area drawn into posteriorly directed angles (Fig. 6, *N.brevipilus*), base of anophoric process stout	***Nannocorisbrevipilus* sp. n.**
9	Glabrous cuticle on metapleuron restricted to ventral margin of scent-gland groove (Leon and Weirauch 2017: Fig. 4, left panel)	**Schizoptera (Schizoptera) henryi sp. n.**
–	Glabrous cuticle surrounding the scent-gland groove extending to or beyond the midline of the metapleuron (Leon and Weirauch 2017: Fig. 4, right panel)	**9**
10	Subgenital plate with 2 small laterad-projecting, slender and acute processes (Fig. 9A)	**Schizoptera (Cantharocoris) bispina McAtee and Malloch**
–	Subgenital plate with large lateral process posteriorly beset with tuft of flattened and long setae and smaller acute posteriad oriented process (Fig. 9D)	**Schizoptera (Cantharocoris) rileyi sp. n.**

### Hypselosomatinae Esaki & Miyamoto, 1959

#### Glyptocombus

Taxon classificationAnimaliaHemipteraSchizopteridae

Heidemann, 1906

**Figure 1:**
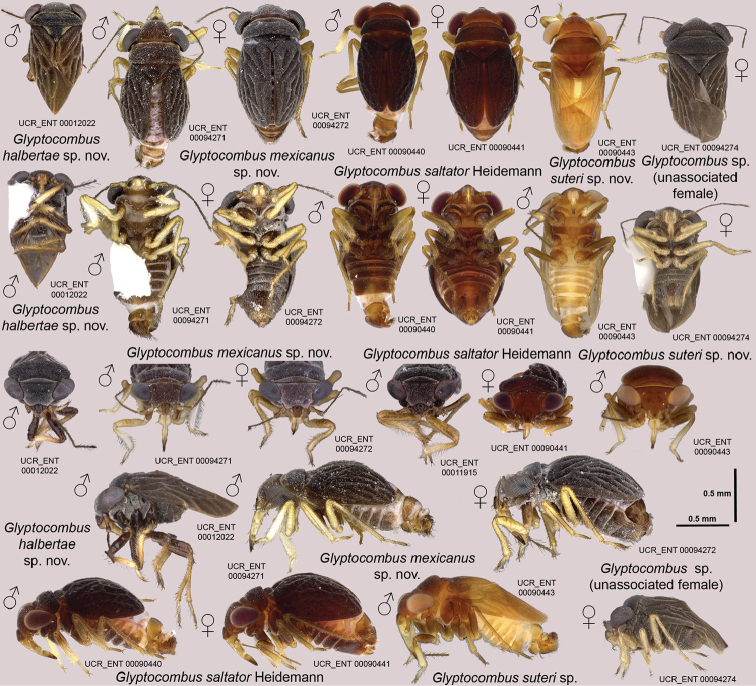
Habitus images of *Glyptocombus* spp. in dorsal, ventral, frontal and lateral views.

**Figure 2.**
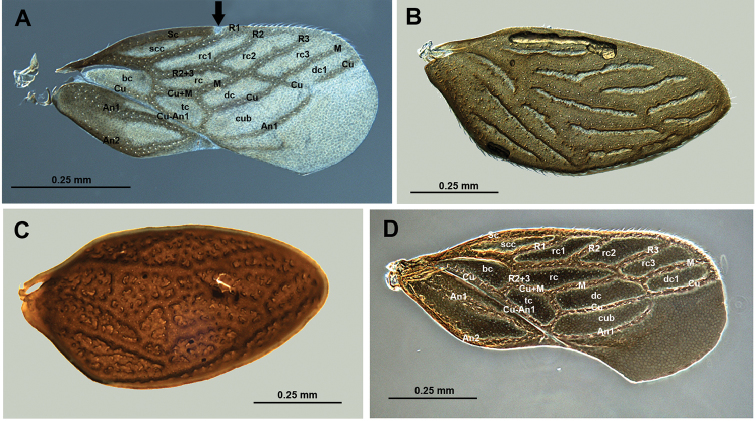
Forewings of males of *Glyptocombus* spp. **A***Glyptocombushalbertae* (UCR_ENT 00012051) **B***G.mexicanus* (UCR_ENT 00094271) **C***G.saltator* (UCR_ENT 00090440) **D***G.suteri* (UCR_ENT 00090443).

**Figure 3.**
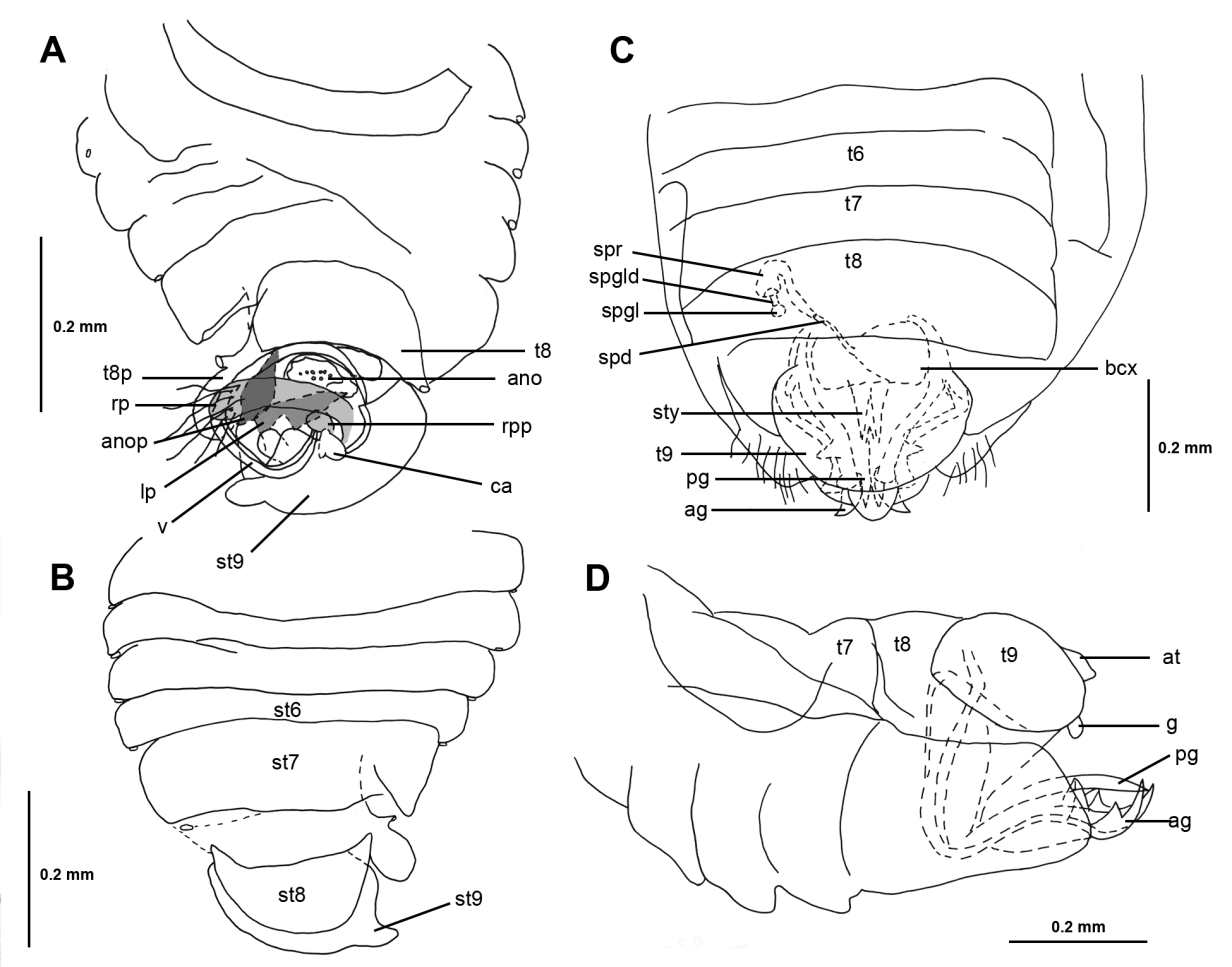
Male genitalia of *G.halbertae* (UCR_ENT 00012051) in **A** dorsal and **B** ventral view, and female genitalia of *G.saltator* (UCR_ENT 00090441) in **C** dorsal and **D** lateral view.

Figures 1 , 2 , 3 , 10

##### Type species.

*Glyptocombussaltator* Heidemann, 1906

##### Revised diagnosis.

Recognized among genera of New World Hypselosomatinae by dense setation on all surfaces of the tibia, extending from base to apex, first labial segment not dorsally expanded, and third and fourth labial segments without processes.

##### Revised description.

**Male** (Figure 1): macropterous or with elytriform forewing, length: 1.14–1.74 mm; body ovoid. **Coloration**: general coloration tan to black. **Surface and Vestiture**: scape with 5 setae, clypeus with 3 long, stout setae, buccula and first labial segment each with pair of stout ventrolateral setae, second labial segment with pair of lateral setae (Figure 1), long, stout seta dorsoapically on third tarsal segment of all legs, anophore without distinctive setae (Figure 3A). **Structure: Head**: first labial segment not enlarged, clypeus simple. **Thorax**: tarsal formula 2-3-3, parempodia long and thin, bladder-like arolium absent. **Abdomen**: abdomen with 6 spiracles on left and 5 on right side, right side of tergum 8 with small projection containing spiracle, left side with large mitten-shaped projection with spiracle and long seta, sterna 7 and 8 distinct, posterior margin of sternum 7 simple, left side with triangular flap, sternum 8 rectangular-ovoid (Figure 3B). **Genitalia** (Figure 3A): right paramere long, stout with rounded apex and short, rounded process at base, left paramere scapula-shaped with large thumb-shaped projection, vesica of average length with one coil and small rounded conjunctival appendage at base, anophoric process crab claw-shaped, extending posteriorly on left side.

**Female** (Figure 1): with elytriform forewing, length: 1.42–1.51 mm; body ovoid. **Coloration**: general coloration tan to black. **Surface and vestiture**: scape with 5 setae, setae on clypeus, buccula and first and second labial segments as in male. **Structure. Head**: first labial segment and clypeus as in male. **Thorax**: tarsal formula 2-2-3, pretarsus as in male (Figure 1). **Genitalia** (Figure 3C, D): anterior gonapophysis with 3 teeth, without subapical serration, posterior gonapophysis with two teeth, median styloid bifurcate, gonoplac small, oriented ventrad, spermathecal gland spherical; spermathecal gland duct straight, spermathecal reservoir globular with one bend, spermathecal duct short and relatively straight.

##### Notes.

This is the only genus of Hypselosomatinae with almost exclusive Nearctic distribution based on locality records of the single previously described and three new species, as well as one female specimen from Chiapas that represents an undescribed species. Specimens have been collected using Berlese extraction, hand collecting, yellow pan traps, pitfall traps (single and array, normal and dung), sifting leaf litter, suction traps, and UV lighting. Extracted substrates included beech humus, bottomland hardwood remnant, fallen leaves, “rubbish,” earth, and the forest floor of Palmetto-gumbo limbo upland.

#### Glyptocombus halbertae sp. n.

Taxon classificationAnimaliaHemipteraSchizopteridae

http://zoobank.org/6197776D-7A16-45A3-9823-1742B315B1E0

Figures 1 , 2 , 3 , 10

##### Material.

***Type material.* Holotype**: male: USA: Florida: Collier Co.: Florida: Collier Co. Immokalee, 26.41853°N 81.41741°W, 04 Jul 2013 - 11 Jul 2013, Susan Halbert (UCR_ENT 00012022) (FSCA). **Paratype**: USA: Florida: Collier Co.: Florida: Collier Co. Immokalee, 26.41853°N 81.41741°W, 26 Jun 2014 - 03 Jul 2014, ED_4274, Susan Halbert, 1 male (UCR_ENT 00012051) (FSCA).

##### Diagnosis.

Recognized among species of *Glyptocombus* by the macropterous male, contrasting dark brown and pale legs, desclerotized portion of C+Sc vein basal to junction with R1, Cu touching M at basal corner of dc1, part of Cu distal to tc s-shaped, R2 slightly sigmoid, rc and dc approx. as long as bc (Figure 2A). Most similar to *G.suteri* based on wing type in males, but differentiated by details of the wing venation.

##### Description.

**Male** (Figure 1): macropterous, length: 1.23 mm; body ovoid. **Coloration**: head, pronotum, and forewings dark brown to black, scutellum dark brown with pale lateral and apical marks, coxae, femora, and base of tibiae dark brown, apex and base of tarsi pale to yellow, pretarsi brown, genital capsule and genitalia dark brown. **Surface and Vestiture**: in frontal orientation forefemur without anterior stout, long seta, hind tibia with 5 erect medium-length stout setae ventrally on distal half, second tarsomere of hind leg with stout seta anteroventrally (Figure 1). **Structure. Head**: disc steeply decurrent anteriorly. **Thorax**: ratio of pronotal collar length to pronotum length 0.23, collar depressed below rest of pronotum (Figure 1), postnotum subrectangular (i.e., freely projecting portion is straight along most of posterior margin, curving only laterally, as in *Rectilamina* Hill; this can be observed only when specimen is in ethanol and wings have been moved aside or removed), ratio of height of forefemur to length of forefemur 0.24, ratio of length of hind tibia to width of pronotum 0.88, venation of macropterous forewing as in Figure 2A, desclerotized portion of C+Sc vein basal to junction with R1, Cu touching M at basal corner of dc1, part of Cu distal to tc s-shaped, R2 slightly sigmoid, rc and dc approx. as long as bc (Figure 2A). **Abdomen and genitalia**: as in genus description (Figure 3A, B).

**Female.** Unknown.

##### Etymology.

Named for the collector of both known specimens of this species, Susan Halbert.

##### Distribution.

Known only from Collier County in Florida (USA).

#### Glyptocombus mexicanus sp. n.

Taxon classificationAnimaliaHemipteraSchizopteridae

http://zoobank.org/728E6BFD-6C7C-4A3C-A0B7-89E7F1A60338

Figures 1 , 2 , 3 , 10

##### Material.

***Type material.* Holotype**: male: MEXICO: Quintana Roo: Res. Ecologica El Eden 25km NNE Leona Vicario, 21.21667°N 87.18333°W, 13 Oct 1998 - 28 Oct 1998, A. Blanco (UCRC_ENT 00038403) (UCR). **Paratypes**: Tamaulipas: Estacion Biol. Los Cedros, Gomez Farias, 22.88629°N 99.0255°W, 350 m, 26 Jul 1993 - 30 Jul 1993, E. G. Riley, 1 male (UCR_ENT 00094271), 1 female (UCR_ENT 00094272) (TAMU); 18 Jul 1994 - 19 Jul 1994, J. Cook & E. Riley, 1 male (UCR_ENT 00094275) (TAMU). Estacion Biol. Los Cedros, Gomez Farias, 22.88621°N 99.0255°W, 18 Jul 1994 - 22 Jul 1994, Cook, Ganaway & Riley, 1 female (UCR_ENT 00094276) (TAMU).

##### Diagnosis.

Distinguished among species of *Glyptocombus* by dark brown coloration, elytriform forewings in both sexes, forewing veins wider than cells, absence of areoles in forewing cells, and all legs with tibia and femora pale (Figure 1). Most similar to *G.saltator* based on wingtype in both males and females, but differentiated by much wider veins.

##### Description.

**Male** (Figure 1): with elytriform forewing, length: 1.18–1.74 mm; body ovoid. **Coloration**: head and body dark brown to black, coxae light brown, rest of legs pale to yellow, genital capsule and genitalia light to dark brown. **Surface and Vestiture**: in frontal orientation forefemur without anterior stout seta, hind tibia withtwoerect medium-length setae ventrally on distal half, second tarsomere of hind leg with stout seta anteroventrally (Figure 1). **Structure. Head**: disc declivous. **Thorax**: ratio of pronotal collar length to pronotum length 0.30–0.32, pronotal collar slightly depressed below pronotum (Figure 1), posterior margin of postnotum curved, ratio of height of forefemur to length of forefemur 0.20–0.23, ratio of length of hind tibia to width of pronotum 1.00, wing venation as in Figure 2B, forewing elytriform, with veins wider than cells. **Abdomen and genitalia**: as in genus description (Figure 3A, B).

**Female** (Figure 1): with elytriform forewing, length: 1.51 mm; body ovoid. **Coloration**: similar to male. **Surface and vestiture**: forefemur with anterior stout seta, hind tibia with four erect medium-length macrosetae ventrally on distal half, second hind tarsomere with two stout setae anteroventrally (Figure 1), entire surface of sternum 7 shiny with long setae (Figure 1). **Structure. Head**: disc decurrent anteriorly. **Thorax**: similar to male, but ratio of pronotal collar length to pronotum length 0.28, collar slightly depressed below pronotum (Figure 1), postnotum short and rectangular, ratio of height of forefemur to length of forefemur 0.26, ratio of length of hind tibia to width of pronotum 1.00, forewing similar to male. **Abdomen and Genitalia**: as in genus description (Figure 3C, D).

##### Etymology.

Named for the country of origin.

##### Distribution.

Specimens of this species have been collected in Quintana Roo and Tamaulipas in Mexico.

#### Glyptocombus saltator

Taxon classificationAnimaliaHemipteraSchizopteridae

Heidemann, 1906

Figures 1 , 2 , 3 , 10

##### Material.

***Type material***. **Holotype**: male: Maryland: Montgomery Co.: Plummers Island, 38.97049°N 77.1763°W, 4.10.05, DH Clemons (UCR_ENT 00028583) (USNM). Paratypes: USA: Maryland: Montgomery Co.: Plummers Island, 38.97049°N 77.1763°W, 29 Sep 1905, DH Clemons, 1 female Paratype No 9785 U.S.N.M.(USNM).

##### Diagnosis.

Recognized among species of *Glyptocombus* by reddish-brown coloration, elytriform forewings in both sexes, forewing veins narrower than cells, and areolate forewing cells (Figure 1). Most similar to *G.mexicanus* based on wingtype in both males and females, but differentiated by much narrower veins.

##### Revised description.

**Male** (Figure 1): with elytriform forewing, length: 1.14–1.52 mm; body ovoid. **Coloration**: head and body reddish dark brown, legs light brown with apex of foretibiae light, genital capsule and genitalia light to dark brown. **Surface and vestiture**: in frontal orientation forefemur without anterior stout seta, hind tibia with four erect medium-length stout setae ventrally on distal half, second tarsomere of hind leg with stout seta anteroventrally. **Structure. Head**: disc decurrent anteriorly. **Thorax**: ratio of pronotal collar length to pronotum length 0.25–0.35, collar slightly depressed below pronotum (Figure 1), posterior margin of postnotum curved in medially around contour of scutellum, ratio of height of forefemur to length of forefemur 0.20–0.23, ratio of length of hind tibia to width of pronotum 0.94–1.16, venation of elytriform forewing as in Figure 2A, veins narrower than cells, cells areolate. **Abdomen and genitalia**: as in genus description (Figure 3A, B).

**Female** (Figure 1): elytrous, length: 1.46–1.49 mm; body ovoid. **Coloration**: similar to male. **Surface and vestiture**: in frontal orientation forefemur without anterior stout seta, hind tibia with four erect medium-length stout setae ventrally on distal half, second hind tarsomere with stout seta anteroventrally, sternum 7 with posteriad-pointing medial U-shaped shiny area with long setae. **Structure. Head**: disc steeply declivous anteriorly. **Thorax**: similar to male, ratio of pronotal collar length to pronotum length 0.26–0.29, collar slightly depressed below pronotum (Figure 1), posterior margin of postnotum curved posteriorly, ratio of height of forefemur to length of forefemur 0.14–0.24, ratio of length of hind tibia to width of pronotum 0.88–1.09, elytriform forewing as in male. **Abdomen and genitalia**: as in genus description (Figure 3C, D).

##### Notes.

A redescription of this species is included to achieve more consistent treatments for species of Old World (Hill 1984, 1987, 1991, 2013) and New World Hypselosomatinae (Carpintero and Dellapé 2006, Hoey-Chamberlain and Weirauch 2016).

##### Distribution.

Specimens of this species have been collected in Maryland, Georgia, Oklahoma, Texas, and Mississippi (USA).

##### Other material examined.

Georgia: Clarke Co.: Whitehall Forest, 33.90694°N 83.35722°W, 07 May 1977 - 12 May 1977, R. Turnbow, 1 male (AMNH_IZC 00150699) (AMNH); 09 Jul 1977 - 15 Jul 1977, R. Turnbow, 1 male (UCR_ENT 00011913) (FSCA); 24 Jul 1977 - 31 Jul 1977, R. Turnbow, 1 adult sex unknown (UCR_ENT 00011914) (FSCA). Glynn Co.: Jekyll Island, 31.06833°N 81.41361°W, 18 Aug 1965, W. R. Suter, 2 females (UCR_ENT 00090439 and UCR_ENT 00090441), 1 male (UCR_ENT 00090440) (FMNH). Mississippi: Pontotoc Co.: Ecru, 34.35316°N 89.02311°W, 07 May 1980, W. H. Cross, 2 adults sex unknown (UCR_ENT 00028695, UCR_ENT 00028697) (USNM); 08 May 1980, W. H. Cross, 1 adult sex unknown (UCR_ENT 00028696) (USNM). Oklahoma: Latimer Co.: Latimer County, no specific locality, 34.83503°N 95.31025°W, May 1991, Karl Stephan, 1 male (UCR_ENT 00011915) (FSCA); May 2002, K. Stephan, 1 male (UCR_ENT 00094270) (TAMU). Texas: Wood Co.: 3 miles WSW Hainsville, 32.70833°N 95.41°W, 28 Apr 2000 - 30 Apr 2000, M. Yoder, 1 male (UCR_ENT 00094273) (TAMU).

##### Specimens from literature.

USA: Arkansas: Chicot Co.: 0.2 mile east of Lake Chicot State Park, 33.37194°N 91.19578°W, 22 Jan 1977, R. G. Chenowith, 1 male (AMNH). Cross Co.: Village Creek State Park, cabin area, 35.16824°N 90.72144°W, 07 Feb 1987, C. E. Carlton, 1 male (AMNH). District of Columbia: no specific locality, 38.90719°N 77.03687°W, Jan or Jun 1879, Theo. Pergande, 1 adult sex unknown (USNM). Maryland: Montgomery Co.: Plummers Island, 38.97049°N 77.1763°W, 08 Oct 1905, Schwarz, Heidemann, 1 adult sex unknown (USNM); 14 Oct 1906, C. H. T. Townsend, 1 adult sex unknown (USNM). Virginia: Northampton Co.: Savage neck dunes natural area preserve, ca 6 km SW of East6lle, 37.32694°N 76.00774°W, 24 Jun 1999 - 28 Jul 1999, Virginia Division of Natural Heritage personnel, 1 male (VMNH); 27 Aug 1999 - 23 Sep 1999, Virginia Division of Natural Heritage personnel, 1 male (VMNH); 23 Sep 1999 - 28 Oct 1999, Virginia Division of Natural Heritage personnel, 2 males (VMNH). Virginia Beach Co.: Oceana Naval Air Station, City of Virginia Beach, ca. 1 mi/1.6 km SSE jct. U.S. Route 58 and Great Neck Road, 37.32694°N 76.00774°W, 14 Jun 1989 - 28 Jun 1989, K. A. Buhlmann, 2 males (VMNH).

#### Glyptocombus suteri sp. n.

Taxon classificationAnimaliaHemipteraSchizopteridae

http://zoobank.org/EA79FC9B-4E23-4CCF-BF13-D39FC5713734

Figures 1 , 2 , 3 , 10

##### Material.

Type material. Holotype: male: USA: Florida: Dade Co.: Everglades National Park, on side of road from Fla. 27, 25.28662°N 80.89865°W, 18 Jun 1965, W. R. Suter, ED_5198 (UCR_ENT 00090443) (FMNH).

##### Diagnosis.

Recognized among *Glyptocombus* species by males macropterous, general coloration light brown (Figure 1, *G.suteri*), C+Sc not desclerotized basal to junction with R1, Cu separated from M by m-cu cross vein, Cu distal to tc straight, R2 straight, rc and dc longer than bc (Figure 2D). Most similar to *G.halbertae* based on wing type in males, but differentiated by details of wing venation.

##### Description.

**Male** (Figure 1): macropterous, length: 1.47 mm; body ovoid. **Coloration**: head and body tan, legs from middle of tibiae to pretarsus pale. **Surface and Vestiture**: in frontal orientation forefemur without anterior stout, long seta, hind tibia with 5 erect medium-length stout setae ventrally on distal half, second tarsomere of hind leg with stout seta anteroventrally (Figure 1). **Structure. Head**: disc declivous. **Thorax**: ratio of pronotal collar length to pronotum length 0.26, collar at same level as pronotum (Figure 1), postnotum trapezoidal, ratio of height of forefemur to length of forefemur 0.26, ratio of length of hind tibia to width of pronotum 0.94, venation of forewing as in Figure 2D, veins wider than cells. **Genitalia**: as in genus description (Figure 3A, B).

**Female.** Unknown.

##### Etymology.

Named for the collector of the single known specimen of this species.

##### Distribution.

Known from the Everglades National Park in Dade County, Florida (USA).

#### Glyptocombus

Taxon classificationAnimaliaHemipteraSchizopteridae

sp. (unassociated female)

Figures 1 , 10

##### Material.

MEXICO: Chiapas: Ocozocoautla de Espinosa Municipality Co.: Reserva El Ocote, Campo El Ocote, 17.04278°N 93.80778°W, 274 m, 26 Jul 1997 - 29 Jul 1997, Gonzalez, Woolley, & Galdamez, 1 female (UCR_ENT 00094274) (TAMU).

##### Notes.

We have examined one undescribed female specimen of *Glyptocombus* from Chiapas in Mexico that has remained unassociated with male specimens (Figure 1; UCR_ENT 00094274). This specimen differs from females of *Glyptocombusmexicanus* sp. n. in being macropterous (elytriform forewings in males and females of *Glyptocombusmexicanus* sp. n.). Although wing polymorphism is common in Hypselosomatinae, species with elytriform forewings in males have not been documented to contain macropterous females (Hill 1984, Hoey-Chamberlain and Weirauch 2016).

### Schizopterinae Reuter, 1891

#### Corixidea major

Taxon classificationAnimaliaHemipteraSchizopteridae

McAtee & Malloch, 1925

Figures 4 , 5 , 10

##### Material.

***Type material.* Holotype**: male: USA: Tennessee: Montgomery Co.: Clarksville, 36.52972°N 87.35944°W, Aug 1915, G. A. Runner (UCR_ENT 00028582) (USNM).

##### Diagnosis.

Recognized among species of *Corixidea* by the uniformly dark coloration, macropterous forewing without pale band, presence of laterally directed tergum 8 process, and very small and rounded anophoric process.

##### Revised Description.

**Figure 4.**
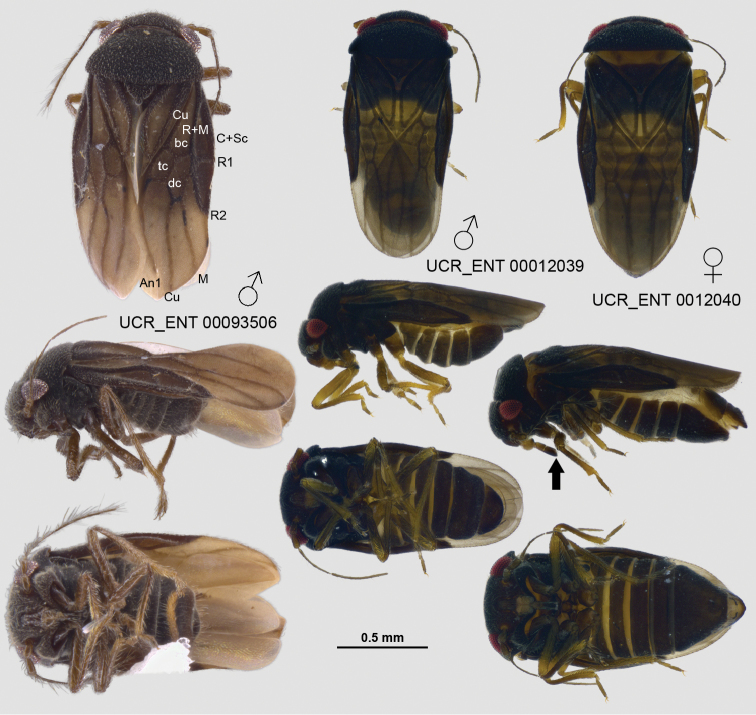
Habitus images of male and female *Corixideamajor* in dorsal, lateral, and ventral view. UCR_ENT 00093506 shows a dry, point-mounted specimen; the other two specimens are ethanol-preserved.

**Male** (Figure 4): macropterous, body length 1.36–1.58 mm; body elongate-oval. **Coloration**: general coloration dark brown to black, head, thorax, and costal area of the forewing dark brown to black, remainder of wing brown to dark brown, antenna and legs uniformly brown, abdomen dark brown to black. **Surface and vestiture**: head, pronotum and wing veins with short to medium-length light brown setae, abdomen densely covered with elongate, light brown setae, wing cells with distinct cell-like sculpture. **Structure: Head**: rounded in ventral view, width of eye ~1/3 width of synthlipsis, ocellus roughly equal in size to one ommatidium, positioned close to margin of compound eye. **Thorax**: pronotum trapezoidal, with posterior margin slightly curved posteriorly, scutellum triangular, with rounded tip, metepisternum with posterior margin rounded, blunt metasternal process widened apically, hind coxa with well-developed adhesive pad, tarsal formula 3-3-3, pretarsus with setiform parempodia, arolia present on fore- and midleg. **Abdomen**: with 5–6 visible sterna corresponding to segments 2+3 (if visible), 4, 5, 6, 7, and 9 (pygophore), sternum 2 longer than preceding sterna, weakly asymmetrical, terga of pregenital abdomen moderately sclerotized, tergum 7 rectangular or weakly trapezoidal, tergum 8 roughly rectangular with process and serrated patch on left side, process of tergum 8 with narrow base and widened apex, aligned with serrated patch on tergum, bearing spinous process. **Genitalia**: pygophore weakly asymmetrical, dorsoventrally flattened, with rounded apex, plane of parameres rotated at 90 degrees with respect to longitudinal body axis, right paramere straight, with robust base and elongate stem, left paramere much smaller than right, roughly as long as wide, aedeagus devoid of large conjunctival appendages, vesica forming 1¼ coils, with long slender process at midpoint of vesica, anophore well sclerotized and with small rounded process.

**Female** (Figure 4): macropterous, body length 1.42 mm, elongate-oval. **Coloration, surface and vestiture**: as in male. **Structure**: as in male but tarsal formula 2-2-3, arolia absent. **Abdomen**: with 4–5 visible sterna corresponding to segments 2+3 (if visible), 4, 5, 6, and 7; sternum 7 much longer than preceding sterna, symmetrical. **Genitalia**: ovipositor vestigial, spermathecal reservoir comma-shaped, spermathecal duct slightly longer than width of tergum 7, connected to left side of bursa copulatrix.

##### Notes.

Distinguished from other US Schizopteridae by a combination of relatively large size, blunt 3-segmented labium, absence of pronotal collar, presence of two triangular cells on costal margin of forewing (bordered by C+Sc, R+M, and R1, and C+Sc, R1, and R2), dorso-ventrally flattened pygophore with rounded apex, very long right paramere, and vesica with long subapical process. The genus *Corixidea* is a part of the *Corixidea* genus group, informally created by Emsley (1969), which also includes *Hoplonannus* McAtee & Malloch, 1925, *Membracioides* McAtee & Malloch, 1925, *Oncerodes* Uhler, 1894, *Voccoroda* Wygodzinsky, 1950, and *Voragocoris* Weirauch, 2012. Monophyly of the group is supported by the blunt labium, absence of a pronotal collar, venational similarities, and by limited molecular data when representatives of three genera were analyzed (Knyshov et al. 2016). However, generic limits that were proposed based on morphological features are unclear when undescribed diversity of the group and the poorly described original species are considered. We are currently working on a revision of the *Corixidea* genus group (Knyshov et al. unpublished), which will contain a comprehensive diagnosis of *C.major* in view of potential taxonomic changes within the group.

##### Other specimens examined.

USA: Florida: Alachua Co.: Gainesville, 29.63527°N 82.37111°W, 24 m, 12 Jun 1966 - 15 Jun 1966, Ladonia O’Berry, 1 female (UCR_ENT 00011922) (FSCA). Broward Co.: Everglades National Park, 25.4775°N 80.96085°W, 24 Aug 1949 - 25 Aug 1949, H. S. Dybas, 1 male (UCR_ENT 00090856) (FMNH). Collier Co.: Florida: Collier Co. Immokalee, 26.41853°N 81.41741°W, 24/IV/2014-1/IV/2014, Susan Halbert, 4 males (UCR_ENT 00012031, UCR_ENT 00012032) (FSCA); 07 Jun 2007 - 14 Jun 2007, Selina Estrada, 1 female (UCR_ENT 00011920) (FSCA); 15 Mar 2012 - 22 Mar 2012, Scott Croxton, 1 female (UCR_ENT 00011918) (FSCA); 08 Nov 2012 - 15 Nov 2012, Scott Croxton, 1 female (UCR_ENT 00011919) (FSCA); 21 Feb 2013 - 28 Feb 2013, Scott Croxton, 1 male (UCR_ENT 00011921) (FSCA); 02 Jan 2014 - 09 Jan 2014, Susan Halbert, 1 male (UCR_ENT 00011867) (FSCA). Immokalee, 26.40611°N 81.41389°W, 13 Jul 2013 - 20 Jul 2013, Susan Halbert, 1 male (UCR_ENT 00011912) (FSCA); 20 Jul 2013 - 27 Jul 2013, Susan Halbert, 2 males (UCR_ENT 00011910, UCR_ENT 00011911) (FSCA); 14 Oct 2013 - 21 Nov 2013, Susan Halbert, 1 female (UCR_ENT 00011868) (FSCA); 07 Nov 2013 - 14 Nov 2013, Susan Halbert, 2 males (UCR_ENT 00011857), 1 female (UCR_ENT 00011858) (FSCA); 05 Dec 2013 - 12 Dec 2013, Susan Halbert, 1 female (UCR_ENT 00011866) (FSCA); 16 Jan 2014 - 23 Jan 2014, Susan Halbert, 1 male (UCR_ENT 00011864) (FSCA); 30 Jan 2014 - 06 Feb 2014, Susan Halbert, 1 male (UCR_ENT 00011865) (FSCA); 13 Mar 2014 - 20 Mar 2014, Susan Halbert, 1 female (UCR_ENT 00012026) (FSCA); 20 Mar 2014 - 22 Mar 2014, Susan Halbert, 2 males (UCR_ENT 00012027, UCR_ENT 00012028) (FSCA); 03 Apr 2014 - 10 Apr 2014, Susan Halbert, 1 male (UCR_ENT 00012029), 1 female (UCR_ENT 00012030) (FSCA); 01 May 2014 - 08 May 2014, Susan Halbert, 5 males (UCR_ENT 00012033-UCR_ENT 00012037) (FSCA); 22 May 2014 - 29 May 2014, Susan Halbert, 1 male (UCR_ENT 00012038) (FSCA); 29 May 2014 - 05 Jun 2014, Susan Halbert, 1 female (UCR_ENT 00012041), 1 male (UCR_ENT 00012042) (FSCA); 05 Jun 2014 - 12 Jun 2014, Susan Halbert, 1 male (UCR_ENT 00012039), 1 female (UCR_ENT 00012040) (FSCA); 03 Jul 2014 - 10 Jul 2014, Susan Halbert, 2 females (UCR_ENT 00012043, UCR_ENT 00012044) (FSCA); 10 Jul 2014 - 17 Jul 2014, Susan Halbert, 4 males (UCR_ENT 00012045-UCR_ENT 00012047, UCR_ENT 00012049), 1 female (UCR_ENT 00012048) (FSCA). Highlands Co.: Parker Islands 7 mi. S.E. of Lake Placid, 27.24476°N 81.29812°W, 13 Jun 1955, H. S. Dybas, 1 female (UCR_ENT 00090857) (FMNH). Hillsborough Co.: Hillsborough River State Park, 28.10735°N 82.27178°W, 07 Feb 1958, F. W. Mead, 1 female (UCR_ENT 00011923) (FSCA). Louisiana: Vermilion Co.: Gueydan, 30.03059°N 92.50833°W, 01 Jul 1925, E. R. Kalmbach, 1 male (UCR_ENT 00026654) (USNM). Oklahoma: Latimer Co.: Latimer County, no specific locality, 34.83503°N 95.31025°W, Jul 1989, Karl Stephan, 2 females (UCR_ENT 00011924, UCR_ENT 00011925), 1 male (UCR_ENT 00011926) (FSCA); Jun 2002, K. Stephan, 2 males (UCR_ENT 00093519, UCR_ENT 00093517) (TAMU); Jul 2002, K. Stephan, 3 females (UCR_ENT 00093528, UCR_ENT 00093527, UCR_ENT 00093510), 4 male (UCR_ENT 00093512, UCR_ENT 00093511, UCR_ENT 00093508, UCR_ENT 00093509) (TAMU); Aug 2002, K. Stephan, 1 female (UCR_ENT 00093516), 4 males (UCR_ENT 00093518, UCR_ENT 00093505-UCR_ENT 00093507) (TAMU). Texas: Brazos Co.: College Station, 30.62778°N 96.33417°W, 03 May 1978 - 09 May 1978, J. A. Jackman, Light Trap, 1 male (UCR_ENT 00094213) (TAMU). College Station, Lick Creek Park, 30.57755°N 96.29052°W, 04 Oct 1987 - 18 Oct 1987, R. Wharton, 1 female (UCR_ENT 00094184) (TAMU). Cameron Co.: Sabal Palm Grove Sanctuary, 25.85016°N 97.4244°W, 16 Oct 1993, Backmon, Quinn & Riley, 1 male (UCR_ENT 00094182), 1 female (UCR_ENT 00094183) (TAMU). Wood Co.: Little Sandy National Wildlife Refuge, 32.57611°N 95.24722°W, 19 Sep 1998, J.D. Oswald, 10 males (UCR_ENT 00094214, UCR_ENT 00094215, UCR_ENT 00093514, UCR_ENT 00093520-UCR_ENT 00093526), 3 females (UCR_ENT 00094216, UCR_ENT 00093513, UCR_ENT 00093515) (TAMU).

**Figure 5.**
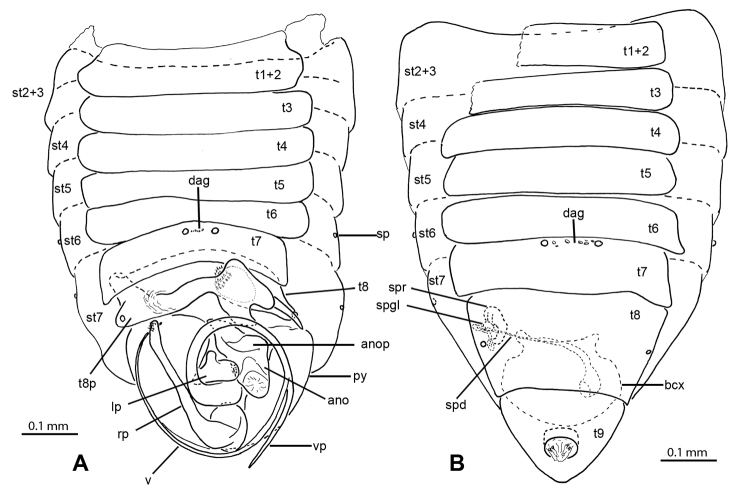
Male and female genitalic features of *Corixideamajor*. A. Male abdomen (UCR_ENT 00012039) in dorsal view; B. Female abdomen (UCR_ENT 00012040) in dorsal view.

### *Nannocoris* Reuter, 1891

#### Nannocoris tuberculiferus

Taxon classificationAnimaliaHemipteraSchizopteridae

Reuter, 1891

##### Notes.

**Nomenclature.**Reuter (1891) described the subgenus Schizoptera (Nannocoris) to accommodate two new species with distinctly elongated heads, Schizoptera (Nannocoris) nebulifera Reuter, 1891 and Schizoptera (Nannocoris) tuberculifera Reuter, 1891. Whereas *Schizoptera* is feminine, *Nannocoris* is masculine, because “coris” is Greek for “bug” and a noun with masculine gender. When McAtee and Malloch (1925) elevated *Nannocoris* to genus level, they failed to adjust the gender of previously described species from feminine to masculine and also treated their newly described species as feminine. Nevertheless, subsequent authors continued to treat *Nannocoris* as feminine. *Nannocorisarenaria* Blatchley, 1926 was corrected to *Nannocorisarenarius* Blatchley, 1926 by Henry (1988) without comment. We here adjust the genders to masculine for all remaining names that were originally treated as feminine and are not patronyms: *N.nebuliferus* Reuter, 1891; *N.tuberculiferus* Reuter, 1891; *N.capitatus* (Uhler, 1894); *N.nasus* McAtee & Malloch, 1925; *N.flavomarginatus* McAtee & Malloch, 1925.

##### Phylogeny and classification.

A recent molecular phylogenetic analysis of *Nannocoris* (Frankenberg et al. 2018) included one North American specimen (UCR_ENT 00094252), a female from Texas that we have assigned to the new species *N.brevipilus* below. This taxon was recovered as part of the *pricei* species group that is diagnosed by the opening of the male-specific vertex gland being located in a posterior position, i.e. posteriorly on the vertex or on the pronotal collar. The opening is more anterior on the head in males of all other *Nannocoris* species we have examined (data not shown; see Figure 1 in Frankenberg et al. [2018] for selected species) and it is absent in some species. Males of *N.arenarius* and the second newly described species, *N.anophorus*, share the pronotal position of the opening of the vertex gland. We therefore tentatively assign all three Nearctic species to the *pricei* species group.

#### Nannocoris anophorus sp. n.

Taxon classificationAnimaliaHemipteraSchizopteridae

http://zoobank.org/4AFF2372-4A4E-4608-8806-0956F2F747FB

Figures 6 , 7 , 10

##### Material.

***Type material*. Holotype**: male: USA: Texas: Hays Co.: 6 mi. NW Dripping Springs, 30.22648°N 98.18493°W, 408 m, 03 Jun 2006 - 30 Jun 2006, E. G. Riley, et al. (UCR_ENT 00094264) (TAMU).

##### Diagnosis.

Recognized among species of *Nannocoris* by relatively short head, ovoid body and forewing shape, yellow costal and posterior claval margins, long setae on forewing veins, vertex gland opening on depression of pronotal collar, short, smoothly rounded vesica with less than one coil, apically bifurcating right paramere, and long and sigmoid anophoric process that reaches anteriorly to terga 5 or 6.

##### Description.

**Figure 6.**
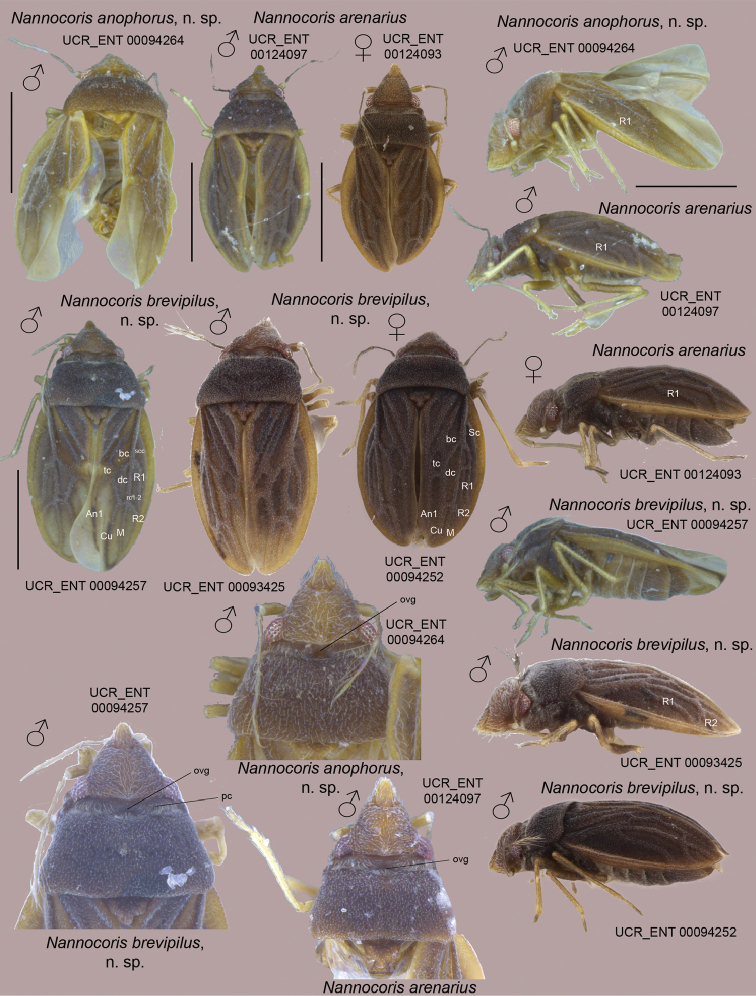
Habitus images of *Nannocoris* spp. in dorsal and lateral views and close-ups of head and pronotum of males to document the unusual position of the vertex gland opening on the pronotal collar.

**Figure 7.**
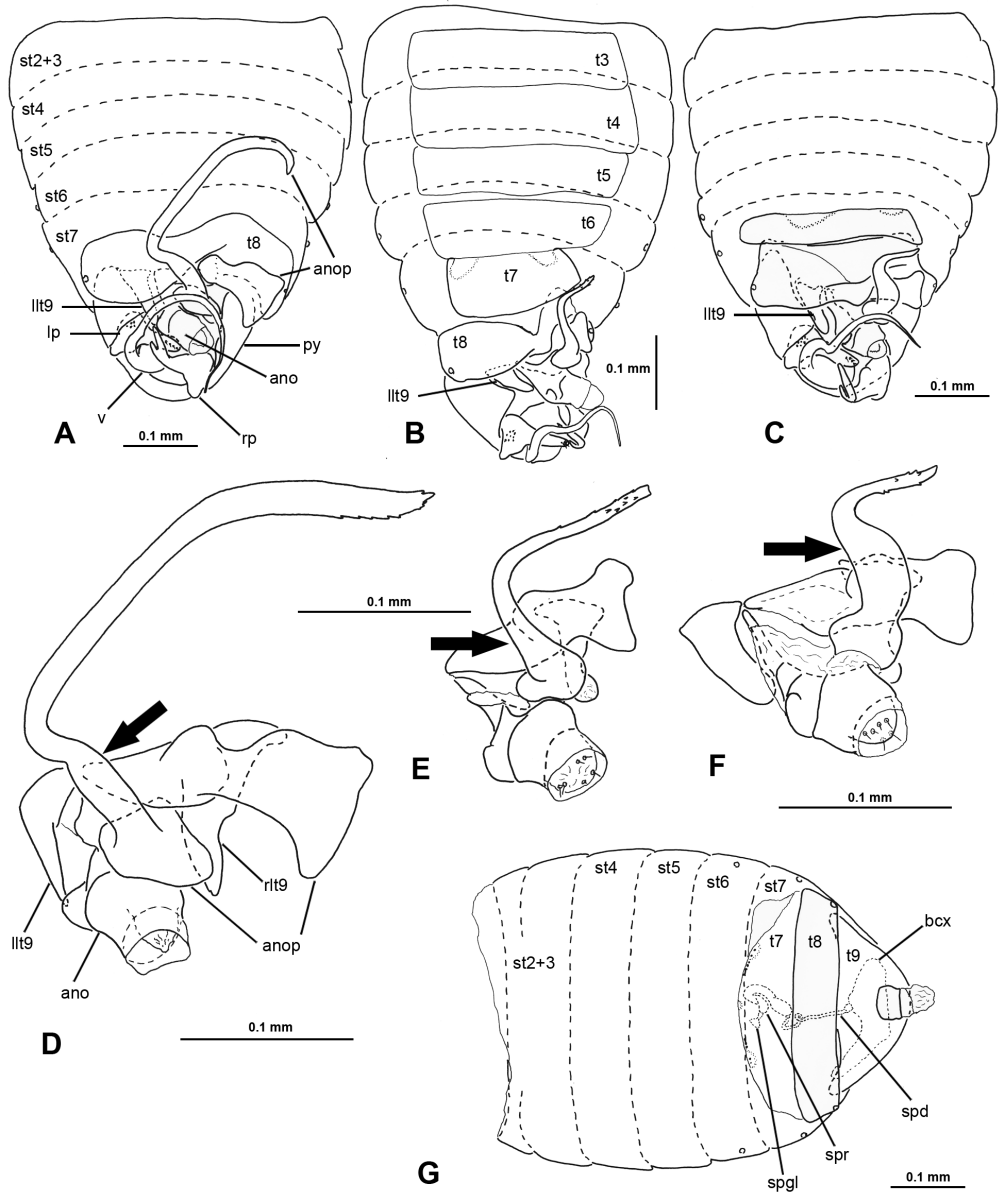
Male and female genitalic features of *Nannocoris* spp. **A–C** Male abdomen: **A***N.anophorus* (UCR_ENT 00094264) **B***N.arenarius* (UCR_ENT 00124097) **C***N.brevipilus* (UCR_ENT 00093425) **D–F** Male anophore with associated sclerites: **D***N.anophorus* (UCR_ENT 00094264) **E***N.arenarius* (UCR_ENT 00124097) **F***N.brevipilus* (UCR_ENT 00094257) **G***N.arenarius*, female abdomen (UCR_ENT 00124095).

**Male** (Figure 6): macropterous, length: 1.16 mm; body ovoid. **Coloration**: general coloration light brown, with head yellowish brown, lighter colored anteriorly, scutellum and costal and posterior claval margins yellow, legs pale yellow (Figure 6). **Surface and vestiture**: forewing veins with long white setae, pronotum and head with dense, short, recumbent white setae. **Structure: Head**: moderately elongate, ca. as high as long, labium very slender, barely surpassing posterior margin of pronotal collar, eye small, ~1/6 of greatest head width. **Thorax**: opening of vertex gland medially on pronotal collar, opening large (Figure 6), forewing macropterous (Figure 6), costal margin slightly explanate, R1 straight, obliquely traversing cells scc and rc1-rc2, merging with Sc close to where R2 reaches wing margin, An1 almost reaching Cu. **Abdomen**: tergum 8 slightly asymmetrical, transverse, right half slightly curved anteriad. **Genitalia** (Figure 7A, D): left laterotergite 9 long and slender, posteriad-oriented spine, right paramere with broad base and narrow, bifurcating apex, left paramere triangular, vesica forming smoothly rounded ¾ loop, anophoric process with anterior broad portion adjacent to tergum 8 and long, sigmoid process reaching anteriorly to approx. level of tergum 5 or 6.

**Female**: Unknown.

##### Etymology.

Named for the long anophoric process that is unusually prominent among species of *Nannocoris*, especially in other species of the *pricei* species group.

##### Notes.

The holotype was collected using a flight intercept trap.

##### Distribution.

Known only from Hays County in Texas.

#### Nannocoris arenarius

Taxon classificationAnimaliaHemipteraSchizopteridae

Blatchley

Figures 6 , 7 , 10

##### Material.

***Type material*. Holotype**: male: USA: Florida: Pinellas Co.: Dunedin, 28.027°N 82.77126°W, Jan 4- Feb 16, W.S. Blatchley (PURC). **Paratypes**: same data as holotype, 11 specimens.

##### Revised diagnosis.

Recognized among species of *Nannocoris* by relatively short head, ovoid body and forewing shape, yellow costal and posterior claval margins, short setae on forewing veins, vertex gland opening on depression of pronotal collar, short and thin, slightly sigmoid vesica, apically bifurcating right paramere, and relatively short s-shaped anophoric process with slender base that reaches anteriorly to tergum 7. Similar to *N.brevipilus*, but distinguished by slender base of anophoric process.

##### Revised description.

**Male** (Figure 6): submacropterous, length: 1.04–1.09 mm, body broadly ovate. **Coloration**: general coloration light brown, with head somewhat lighter, scutellum and costal and posterior claval margins yellow, legs pale yellow with basal ¾ of femora light brown (Figure 6). **Surface and vestiture**: head, pronotum and forewing veins with dense, short, recumbent white setae. **Structure: Head**: moderately elongate, slightly longer than high, labium very slender, reaching to approx. mid coxa, eye small, ~1/6 of greatest head width. **Thorax**: opening of vertex gland medially on pronotal collar, opening large (Figure 6); forewing submacropterous (Figure 6), costal margin slightly explanate, R1 sinuously traversing cells scc and rc1-rc2, merging with Sc proximal to R2 reaching wing margin. **Abdomen**: tergum 8 strongly asymmetrical, much wider in left half, right half narrow and curved anteriad. **Genitalia** (Figure 7B, E): left laterotergite 9 relatively short, laterad-oriented spine, right paramere with narrow, bifurcating apex, left paramere elongate-triangular, vesica sinuous, not forming loop, anophoric process with anterior broad portion adjacent to tergum 8 and s-shaped process reaching anteriorly to posterior margin of tergum 7, base of s-shaped process slender.

**Female** (Figure 6): similar to male, length: 1.00–1.02 mm. **Genitalia**: as in Figure 7G.

##### Notes.

Specimens have been collected from leaf litter associated with *Quercusmyrtifolia* Wild, *Quercuschapmanii* Sarg, and *Panicum* grass; berleseate of dried cattle manure; and flight intercept traps.

##### Distribution.

Specimens examined by us are from Highlands, Pinellas, and Polk Counties in Florida, including specimens collected at the type locality. Specimens from Georgia, North Carolina, and Virginia deposited at NCSU and VMNH were identified as *N.arenarius* by Robert L. Blinn, Richard L. Hoffman, and Steven L. Roble. *Nannocorisanophorus* and *Nannocorisbrevipilus* are currently known only from Texas; the male illustrated by Hoffman et al. (2006) is submacropterous. We therefore assume that the specimens from Georgia, North Carolina, and Virginia are *N.arenarius*, but male genitalic structures should be examined to confirm this hypothesis.

##### Other material examined.

USA: Florida: Highlands Co.: Lake Placid, Archbold Biological Station, 27.188°N 81.337°W, 03 Feb 1984, M. Deyrup, 2 females (UCR_ENT 00124094, UCR_ENT 00124095), 1 male (UCR_ENT 00124097) (ABS); 01 Feb 1986, M. Deyrup, 1 female (UCR_ENT 00124096) (ABS). Pinellas Co.: Dunedin, 28°N 82°W, Dec 1929 - Apr 1930, W. S. Blatchley, 1 male (UCR_ENT 00120010) (NHMUK). Polk Co.: Lake Wales Ridge Forest, 27.66388°N 81.39455°W, 16 Jul 2009, H. Otte, M. Deyrup, N. Deyrup, 1 female (UCR_ENT 00124093) (ABS). Georgia: Bryan Co.: no specific locality, 32.16562°N 82.90008°W, 17 Sep 1974, R. Beshear, 1 female (USNM).

##### Specimens databased from other collections (not examined by us).

North Carolina: Davidson Co.: Davidson, 35.77224°N 80.1878°W, 11 Jul 1976, T. Daggy, 75 males (NCSU_ENT 00216994-NCSU_ENT 00216998) (NCSU). Mecklenburg Co.: Davidson College, Davidson, 35.50173°N 80.84678°W, 839 m, 11 Nov 1955, T. Daggy, 5 males (NCSU). Virginia: Suffolk Co.: South Quay pine barrens, “100 m north of the canal”, ca. 13 km S of Franklin, 36.55843°N 76.90858°W, 02 Jul 2003 - 06 Aug 2003, S.M. Roble, 2 males, 1 female (VMNH); 06 Aug 2003 - 13 Sep 2003, S.M. Roble, 2 males (VMNH).

#### Nannocoris brevipilus sp. n.

Taxon classificationAnimaliaHemipteraSchizopteridae

http://zoobank.org/D041EB3E-C766-4297-AEBF-9853DFF975A2

Figures 6 , 7 , 10

##### Material.

***Type material*. Holotype**: male: USA: Texas: Sabine Co.: Beech Bottom, 9 mi E Hemphill, 31.38226°N 93.70455°W, 05 Jun 1989 - 17 Jun 1989, R. Anderson & E. Morris (UCR_ENT 00094257) (TAMU). Paratype: USA: Texas: Angelina Co.: Angelina Nat’l. Forest ca. 3 mi. NE Rockland, 31.05528°N 94.36833°W, 02 May 1996 – 16 May 1996, Clarke, Menard, & Riley, 1 male (UCR_ENT 00093425) (TAMU).

##### Diagnosis.

Recognized among species of *Nannocoris* by relatively short head, ovoid body and forewing shape, yellow costal and posterior claval margins, short setae on forewing veins, vertex gland opening on depression of pronotal collar, short and thin, slightly sigmoid vesica, apically bifurcating right paramere, and relatively short s-shaped anophoric process with slender base that reaches anteriorly to tergum 7. Similar to *N.brevipilus*, but distinguished by slender base of anophoric process.

##### Description.

**Male** (Figure 6): submacropterous, length: 1.04–1.09 mm, body broadly ovate. **Coloration**: general coloration light brown, head somewhat lighter, scutellum and costal and posterior claval margins yellow, legs pale yellow with basal ¾ of femora light brown (Figure 6). **Surface and vestiture**: head, pronotum and forewing veins with dense, short, recumbent white setae. **Structure: Head**: moderately elongate, slightly longer than high, labium very slender, reaching to ca. midcoxa, eye small, ~1/6 of greatest head width. **Thorax**: opening of vertex gland medially on pronotal collar, opening large (Figure 6), forewing submacropterous (Figure 6), costal margin slightly explanate, R1 sinuously traversing cells scc and rc1-rc2, merging with Sc proximal to R2 reaching wing margin. **Abdomen**: tergum 8 strongly asymmetrical, much wider in left half, right half narrow and curved anteriad. **Genitalia** (Figure 7B, E): left laterotergite 9 relatively short, laterad-oriented spine, right paramere with narrow, bifurcating apex, left paramere elongate triangular, vesica sinuous, not forming loop, anophoric process with anterior broad portion adjacent to tergum 8 and s-shaped process reaching anteriorly to posterior margin of tergum 7, base of s-shaped process slender.

**Female** (Figure 6): similar to male, length: 1.11–1.23 mm, shorter and more ovoid than male, yellow borders of costal and claval margin less pronounced, forewings shorter.

##### Etymology.

Named for the short vestiture on the wings that distinguishes this species from the second species occurring in Texas, *N.anophorus*; a combination of the Latin *brevis* (short) and *pilus* (hair).

##### Notes.

We treat a series of female specimens collected in Bastrop County as conspecific with *N.brevipilus*. We refrain from treating these specimens as paratypes, because no syntopic males are available and females of *N.anophorus* are unknown. We argue that these specimens are unlikely to be conspecific with *N.anophorus* because of the short vestiture, but we cannot exclude the possibility that they are females of a yet undiscovered species of *Nannocoris*.

Males were collected using pitfall and flight intercept traps, females with Berlese extraction.

##### Distribution.

Known from Angelina, Bastrop, and Sabine Counties in Texas.

##### Other material examined.

USA: Texas: Bastrop Co.: Bastrop State Park, 30.11222°N 97.26056°W, 11 Mar 1995, R. Wharton, 6 females (UCR_ENT 00094250-UCR_ENT 00094255), 1 female (UCR_ENT 00094256) (TAMU).

### *Schizoptera* Fieber, 1860

#### Subgenus Schizoptera (Cantharocoris) McAtee & Malloch, 1925

##### Schizoptera bispina

Taxon classificationAnimaliaHemipteraSchizopteridae

McAtee & Malloch, 1925

###### Material.

***Type material.* Holotype**: male: GUATEMALA: Alta Verapaz: Cacao Trece Aguas, 15.4°N 89.75°W, 1906, Schwarz & Barber (UCR_ENT 00028598) (USNM).

###### Revised diagnosis.

Recognized among species of Schizoptera (Cantharocoris) by fairly uniformly light brown coloration and whitish membrane, broad and shallow posterior process on sternum 6, weakly asymmetrical subgenital plate with two small laterad-projecting slender and acute processes (Figure 9A), spine-like right conjunctival appendage and small left conjunctival appendage with three lobes (Figure 9C), long and curved right paramere with abruptly narrowed apex, roughly quadrate left paramere (Figure 9B), and looping vesica moderately slender with 2–3 coils (Figure 9B).

###### Revised description.

**Figure 8.**
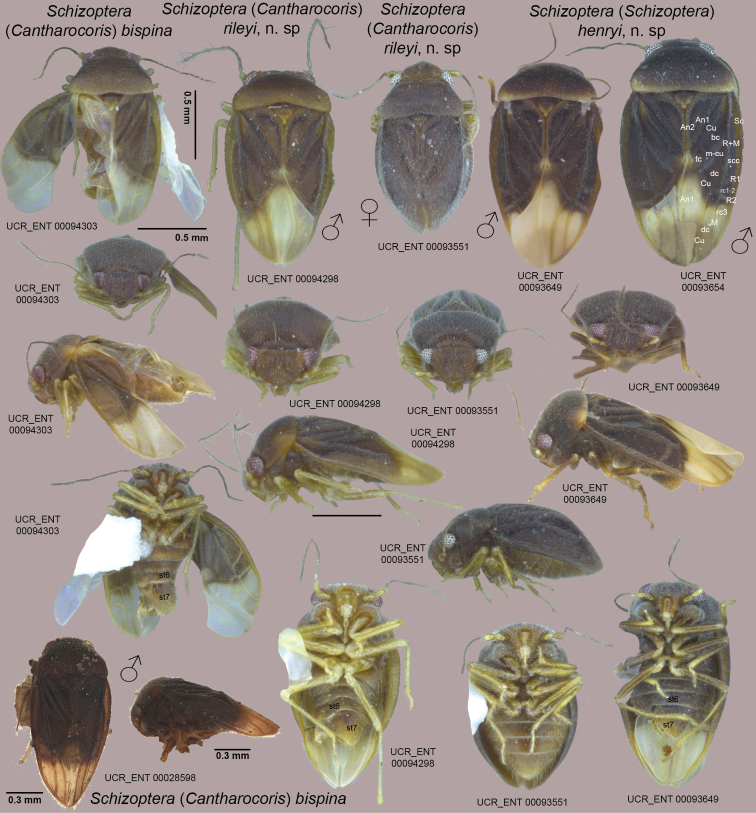
Habitus images of *Schizoptera* spp. in dorsal, frontal, lateral, and ventral views. UCR_ENT 00028598 (lower left corner) is the male holotype of S. (C.) bispina. Scale bar: 0.5 mm, except where indicated otherwise.

**Figure 9.**
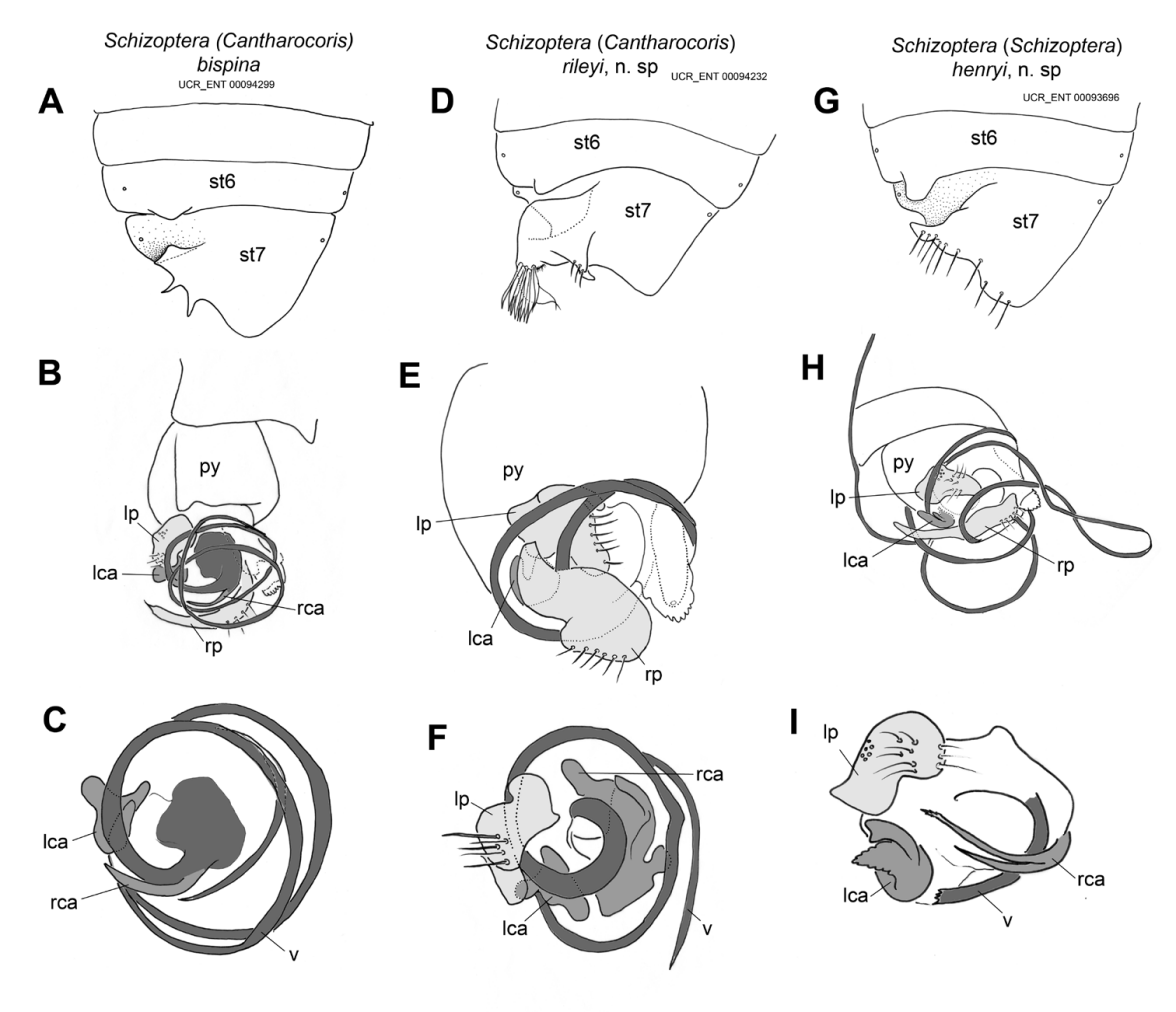
Male genitalic features of *Schizoptera* spp. Top row illustrating ventral view abdominal apex and diagnostic features of sternum 6 and the subgenital plate (sternum 7); middle row showing the pygophore and associated structures in dorsal or dorsolateral view; and bottom row depicting vesica or base of vesica with conjunctival appendages and left parameres after removal of right paramere for S. (C.) rileyi and S. (S.) henryi, and removal of both parameres in S. (C.) bispina.

**Male** (Figure 8): macropterous, length: ~1.3 mm; body broadly ovate. **Coloration** (Figure 8): uniformly light brown except humeral angles and posterior margin of pronotum, costal margin, and scutellum laterally yellow, membrane largely white with narrow proximal boarder dark, vein only slightly darker, legs pale yellow (Figure 8). **Surface and vestiture**: relatively short and dense on head and pronotum, forewing veins with sparse, short setae. **Structure: Head**: triangular in frontal view, slightly wider than high (Figure 8), synthlipsis ~3 times width of eye. **Thorax**: posterior pronotal margin slightly sinuate, R1 distinct, dc1 very slender, especially basally. **Abdomen**: sternum 6 with broad and shallow posterior process, subgenital plate weakly asymmetrical withtwosmall laterad projecting slender and acute processes (Figure 9A). **Genitalia** (Figure 9A–C): right conjunctival appendage spine-like, left conjunctival appendage small, with 3 lobes (Figure 9C), right paramere long and curved, with abruptly narrowed apex, left paramere roughly quadrate (Figure 9B), vesica looping, moderately slender, with 2–3 coils (Figure 9B).

**Female.** One female specimen reported (but not illustrated) by Blatchley (1926) and not examined in our study.

###### Notes.

McAtee and Malloch (1925) described this species from Guatemala based on a single male specimen. They mentioned the slightly yellowish humeral angle, dark color proximally across the membrane, and only slightly notched scutellar apex as characters distinguishing this species from Schizoptera (Cantharocoris) sulcata McAtee & Malloch, 1925, while emphasizing the importance of the shape of the subgenital plate. The characteristic lateral spines on the subgenital plate are shorter in the specimen from Mexico that McAtee and Malloch (1925) considered conspecific with S. (C.) bispina. Blatchley (1926) reported S. (C.) bispina from Florida and provided a redescription. The specimens examined by us are clearly conspecific with those examined and illustrated by Blatchley (1926) based on his fairly detailed description of coloration and drawing of the subgenital plate. No other described species of Schizoptera (Cantharocoris) has a subgenital plate that even remotely resembles the one in S. (C.) bispina. Nevertheless, the distribution range of S. (C.) bispina is much larger than the ranges typically seen in schizopterids. A comprehensive revision of Schizoptera (Cantharocoris) across the Nearctic and Neotropical regions is therefore not unlikely to reveal that the current concept of S. (C.) bispina is a complex of several closely related species.

Blatchley (1926) reported the three specimens he examined as “beaten from Spanish moss,” “sifted from vegetable debris,” and “beaten from sugar cane.” New records indicate that specimens were collected by UV lighting and Malaise trapping.

###### Distribution.

Guatemala, Mexico, and Florida, Louisiana, and Texas in the United States.

###### Other specimens examined.

MEXICO: Tamaulipas: or Unknown Co.: Tampico, 22.2331°N 97.86105°W, December 15, E. A. Schwarz, 1 male (USNM). USA: Florida: Clay Co.: Gold Head Branch St. Park, 29.84638°N 81.96171°W, 07 May 1985, R.W. Jones, 1 male (UCR_ENT 00094299) (TAMU). Pinellas Co.: Dunedin, 28.027°N 82.77126°W, Jan. 19-April 15, Blatchley, 2 male, 1 female (AMNH). Louisiana: Bossier Co.: Bodcau Wdlf. Mgt. Ar., 32.7°N 93.5°W, 22 May 1996, E.G. Riley, 1 male (UCR_ENT 00094303) (TAMU). Texas: Sabine Co.: 9 mi. E. Hemphill, “Beech Bottom,” 31.34135°N 93.6974°W, 22 May 1989 - 04 Jun 1989, R. Anderson & E. Morris, 1 male (UCR_ENT 00093577) (TAMU).

##### Schizoptera rileyi sp. n.

Taxon classificationAnimaliaHemipteraSchizopteridae

http://zoobank.org/1146075C-514D-401C-892D-0BEE52A6D4DC

###### Material.

***Type material*. Holotype**: male: USA: Louisiana: Natchitoches Par Kisatchie Natl. For. Red Dirt W.M.A., 31.35549°N 92.43442°W, 12 Jul 1985, E.G. Riley, 1 male (UCR_ENT 00094298) (TAMU). **Paratypes**: USA: Louisiana: Natchitoches Par Kisatchie Natl. For. Red Dirt W.M.A., 31.35549°N 92.43442°W, 12 Jul 1985, E.G. Riley, 5 males (UCR_ENT 00094301, UCR_ENT 00094308-UCR_ENT 00094311) (TAMU). Natchitoches Park, Kisatchie National Forest, Red Bluff Camp, 31.498°N 93.144°W, 01 Apr 1989, E. Riley & L. Prochaska, 1 female (UCR_ENT 00094315) (TAMU). Texas: Brazos Co.: Koppe’s Bridge, 5 mi SW College Station, 30.58227°N 96.39809°W, 06 Mar 1988, R. Anderson, 1 female (UCR_ENT 00094314) (TAMU); 20 Nov 1993, E. Riley, 1 female (UCR_ENT 00094316), 3 females (UCR_ENT 00094317, UCR_ENT 00094312, UCR_ENT 00094313) (TAMU). Cameron Co.: 10.7 mi. N jct. 106 on FM 2925, 30.85325°N 96.97693°W, 02 Sep 1995 - 03 Sep 1995, E.G. Riley, 1 male (UCR_ENT 00094307) (TAMU). Sabal Palm Grove Ref. (site 1), 25.84851°N 97.41794°W, 03 Sep 2008 - 16 Sep 2008, E.G. Riley & J. King, 1 male (UCR_ENT 00093726) (TAMU). Sabal Palm Grove Ref. (site 2), 25.84851°N 97.41794°W, 03 Oct 2008 - 16 Oct 2008, E. Riley, 1 male (UCR_ENT 00093727) (TAMU); 17 Oct 2008 - 30 Oct 2008, E. Riley, 2 males (UCR_ENT 00093721, UCR_ENT 00093724) (TAMU); 31 Oct 2008 - 02 Jun 2009, E.G. Riley, 1 female (UCR_ENT 00093550) (TAMU). Sabal Palm Grove Sanctuary, 25.85016°N 97.4244°W, 16 Oct 1993 - 17 Oct 1993, H. Blackmon & M. Quinn & E. Riley, 1 female (UCR_ENT 00094306) (TAMU); 02 Sep 1995, E.G. Riley, 1 male (UCR_ENT 00094300) (TAMU). Hidalgo Co.: Santa Ana NWR (site 3) Wildlife Drive, 26.07526°N 98.1388°W, 03 May 2008 - 17 May 2008, E. Riley, 1 female (UCR_ENT 00093549) (TAMU). Kerr Co.: 6.5 mi. SW Hunt, 29.99015°N 99.3874°W, 1960 m, 12 Nov 2005 - 15 Dec 2005, E.G. Riley, 1 female (UCR_ENT 00093551) (TAMU); 24 Feb 2006 - 30 Mar 2006, E.G. Riley, 1 female (UCR_ENT 00094320) (TAMU); 31 Mar 2006 - 27 Apr 2006, E.G. Riley, 2 females (UCR_ENT 00094318, UCR_ENT 00094319) (TAMU); 28 Apr 2006 - 02 Jun 2006, E.G. Riley, 1 female (UCR_ENT 00093548) (TAMU); 01 Jul 2006 - 27 Jul 2006, E.G. Riley, 1 male (UCR_ENT 00093718) (TAMU); 01 Jul 2006 - 27 Jul 2006, E.G. Riley, 4 males (UCR_ENT 00093728, UCR_ENT 00093730, UCR_ENT 00093719, UCR_ENT 00093720), 4 males (UCR_ENT 00094232, UCR_ENT 00093552-UCR_ENT 00093554) (TAMU); 28 Jul 2006 - 01 Sep 2006, E.G. Riley, 1 male (UCR_ENT 00093725) (TAMU); 28 Jul 2006 - 01 Sep 2006, E.G. Riley, 1 male (UCR_ENT 00093729) (TAMU).

###### Diagnosis.

Recognized among species of Schizoptera (Cantharocoris) by uniformly light to yellowish brown coloration, medium-sized posterior process on sternum 6, and subgenital plate with large lateral process posteriorly beset with tuft of flattened and long setae and smaller acute posteriad-oriented process (Figure 9D).

###### Description.

**Male** (Figure 8): macropterous, length: 1.42–1.48 mm; body ovate. **Coloration**: (Figure 8): generally light and yellowish brown, posterior margin of pronotum, costal margin, and scutellum yellow, Cu proximally, An1 on cuneus and An2 lined anteriorly with contrasting dark coloration, membrane white except 3 and dc1 yellowish and apical 1/3 slightly suffused, legs pale yellow (Figure 8). **Surface and vestiture**: long and moderately dense on head, pronotum, and forewing veins. **Structure: Head**: triangular in frontal view, wider than high (Figure 8), synthlipsis slightly less than three times width of eye. **Thorax**: with posterior pronotal margin medially slightly concave, posterior margin of clavus broadly elevated, R1 obsolete, dc1 very slender, width similar throughout. **Abdomen**: sternum 6 with medium-sized posterior process, with large lateral process beset posteriorly with tuft of flattened and long setae and smaller acute posteriad- oriented process (Figure 9D). **Genitalia** (Figure 9D–F): right conjunctival irregularly shaped with two lobes and several ridges, left conjunctival appendage smaller, with three lobes (Figure 9F), right paramere large, broad, with broad, curved apex (Figure 9E), left paramere roughly quadrate with thumb-like process (Figure 9E), vesica looping, fairly stout, with slightly fewer than two coils (Figure 9B).

**Female** (Figure 8): coleopteroid with very narrow membranous border, length: 1.22–1.29 mm; body ovate. **Coloration**: (Figure 8): more uniformly brown compared to male, head slightly paler, legs pale yellow, An2 anteriorly and claval furrow lined with contrasting dark coloration, similar to pattern in male. **Surface and vestiture**: similar to male. **Structure: Head**: triangular in frontal view, approx. as wide as high (Figure 8), synthlipsis slightly more than 3 times width of eye. **Thorax**: pronotum narrower than in male, posterior pronotal margin medially slightly concave, forewing veins obsolete, claval furrow distinct, posterior margin of clavus broadly elevated. **Genitalia**: not examined.

###### Etymology.

Named in honor of Dr. Ed Riley, who collected most of the specimens examined for this study. A noun in genitive case.

###### Notes.

Coleopteroid females evolved several times independently in the Schizoptera genus group (Leon and Weirauch 2017), but female coleopteroidy appears to be especially prevalent in the subgenus Schizoptera (Cantharocoris). Associating conspecific macropterous males and coleopteroid females is often a challenge in *Schizoptera*. Species diagnoses heavily rely on male genitalic features, long series of males and females from a small collection event are rare, and molecular matching of males and females has been done only for a few minute litter bugs (Knyshov et al. 2016). Matching male and female *Schizopterarileyi* was comparatively straightforward because of the long series of specimens of both sexes from across the known distribution range, but also because of the distinctive dark lines on the forewings.

Although the collection method is unknown for the majority of specimens examined, both sexes have been collected using flight intercept traps and some males have been taken at UV light traps.

###### Distribution.

Known only from the U.S., where it has been collected in Natchitoches Parish in Louisiana and Brazos, Cameron, and Hidalgo Counties in Texas.

#### Subgenus Schizoptera (Schizoptera) McAtee & Malloch

##### Schizoptera henryi sp. n.

Taxon classificationAnimaliaHemipteraSchizopteridae

http://zoobank.org/1FE7CD11-1F9D-4DA6-A909-29235C12FB03

###### Material.

***Type material*. Holotype**: male: USA: Texas: Cameron Co.: 1.5 mi. E. jct. FM, 1419 on Hwy 4, E of Brownville, 25.88758°N 97.43592°W, 19 Oct 2002, B. Raber & E. Riley, 1 male (UCR_ENT 00093649) (TAMU). **Paratypes**: MEXICO: Tamaulipas: Estacion Biol. Los Cedros, Gomez Farias, 22.88621°N 99.0255°W, 28 Jul 1993 - 29 Jul 1993, E. Riley & M. Quinn, 1 male (UCR_ENT 00093696) (TAMU). Veracruz: San Andres Tuxtla Co.: Est. Biol. Los Tuxtlas, Vigia Trail, 18.5849°N 95.07393°W, 450 m, 18 Jun 1997, Wilson & Woolley, 1 male (UCR_ENT 00093704) (TAMU). USA: Texas: Cameron Co.: 1.5 mi. E. jct. FM, 1419 on Hwy 4, E of Brownville, 25.88758°N 97.43592°W, 19 Oct 2002, B. Raber & E. Riley, 1 male (UCR_ENT 00093656) (TAMU). Laguna Atascosa NMR (site 1), 26.22375°N 97.35454°W, 06 Feb 2009 - 26 Feb 2009, E.G. Riley & J. King, 1 immature (UCR_ENT 00093555) (TAMU); 23 Apr 2009 - 05 May 2009, E.G. Riley & J. King, 1 male (UCR_ENT 00093664) (TAMU). Sabal Palm Grove, 25.8525°N 97.4175°W, 24 Oct 1992, E. G. Riley, 1 male (UCR_ENT 00093654) (TAMU).

###### Diagnosis.

Recognized among species of Schizoptera (Schizoptera) by fairly uniformly dark brown coloration with contrasting yellow posterior pronotal margin and humeral angle, whitish membrane with apical ½ suffused, and contrasting yellow and brown legs, large posterior process on sternum 6, and subgenital plate with medium-sized finger-shaped lateral process pointing laterad and with distinctive border of stout setae along right margin of subgenital plate (Figure 9G).

###### Description.

**Male** (Figure 8): macropterous, length: 1.51–1.67 mm; body ovate. **Coloration** (Figure 8): general coloration dark brown, posterior margin of pronotum, humeral angles, scutellar margin, and marking distal to apex of clavus yellow, costal and posterior claval margins yellowish brown, membrane white with apical 1/2 slightly suffused, legs with coxae and femora brown, trochanters yellow, tibiae brown proximally and yellow distally, (Figure 8). **Surface and vestiture**: long and moderately dense on head, pronotum, and forewing veins. **Structure: Head**: triangular in frontal view, distinctly wider than high (Figure 8), synthlipsis slightly more than twice width of eye. **Thorax**: posterior pronotal margin almost straight, very weakly concave medially, dc1 of similar width throughout, almost as wide as 3. **Abdomen**: sternum 6 with large posterior process, subgenital plate with medium-sized finger-shaped lateral process pointing laterad and with distinctive border of stout setae along right margin of subgenital plate (Figure 9G). **Genitalia** (Figure 9G–I): right conjunctival appendage with two spines, longer apically, beset with small tubercles, left conjunctival with two lobes (Figure 9I), right paramere long and slender, with gently curving apex (Figure 9H), left paramere roughly triangular with broad apex (Figure 9I,H), vesica looping, very slender, with more than four coils (Figure 9H).

**Female**: Unknown.

###### Etymology.

Named in honor of Dr. Thomas Henry on the occasion of his 70^th^ birthday and in recognition of his outstanding contributions to heteropterology. We also thank Tom for advancing our understanding of the little-studied Schizopteridae in the United States by keeping his dedicated eye open for these tiny bugs and publishing updated distribution records. A noun in genitive case.

###### Distribution.

Known from Tamaulipas and Veracruz in Mexico and Cameron County in Texas in the U.S.

###### Notes.

This species is unique among *Schizoptera* in the U.S. in having brown and yellow contrasting legs, in addition to the distinctive features of the male abdomen and genitalia.

**Figure 10.**
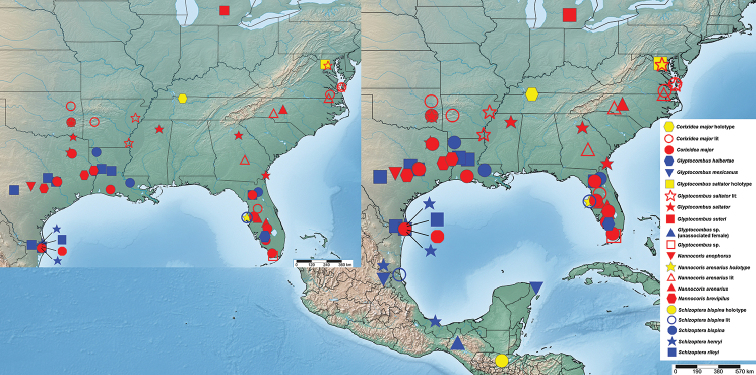
Distribution records of Schizopteridae in the United States and records of selected species from Mexico and Guatemala. Holotype localities of previously described species are indicated in yellow, records from the literature indicate as unfilled symbols, and records reported for the first time in this publication as filled blue or red symbols.

## Supplementary Material

XML Treatment for Glyptocombus

XML Treatment for Glyptocombus halbertae

XML Treatment for Glyptocombus mexicanus

XML Treatment for Glyptocombus saltator

XML Treatment for Glyptocombus suteri

XML Treatment for Glyptocombus

XML Treatment for Corixidea major

XML Treatment for Nannocoris tuberculiferus

XML Treatment for Nannocoris anophorus

XML Treatment for Nannocoris arenarius

XML Treatment for Nannocoris brevipilus

XML Treatment for Schizoptera bispina

XML Treatment for Schizoptera rileyi

XML Treatment for Schizoptera henryi
